# Comparative Analysis of Bread Quality Using Yeast Strains from Alcoholic Beverage Production

**DOI:** 10.3390/microorganisms12122609

**Published:** 2024-12-17

**Authors:** Anqi Chen, Chenwei Pan, Jian Chen

**Affiliations:** 1 Science Center for Future Foods, Jiangnan University, Wuxi 214122, China; 6230210021@stu.jiangnan.edu.cn; 2 School of Biotechnology and Key Laboratory of Industrial Biotechnology of Ministry of Education, Jiangnan University, Wuxi 214122, China; 3 Jiaxing Institute of Future Food, Jiaxing 314050, China; 4 State Key Laboratory of Food Science and Resources, Jiangnan University, Wuxi 214122, China

**Keywords:** commercial yeast strain, bread, hardness, residual sugar, organic acid

## Abstract

The impact of yeast strain selection on bread quality was evaluated using a range of commercial *Saccharomyces cerevisiae* strains, typically employed in various alcoholic beverage productions, to determine their effectiveness in bread making. The final products made from these strains were compared to bread produced using the commercial baker’s strain *S. cerevisiae* ACY298. Key parameters, including specific volume, hardness, pH, residual sugars, and organic acids, were thoroughly assessed. Among the strains tested, *S. cerevisiae* ACY158 produced bread with a specific volume of 5.0 cm^3^/g and a Euclidean distance of 0.895, closely resembling ACY298. In contrast, *S. cerevisiae* ACY9, with a specific volume of 1.1 cm^3^/g and the highest Euclidean distance of 6.878, exhibited the greatest deviation from ACY298, suggesting it may be less suitable for traditional bread production. Furthermore, ACY158 displayed a balanced organic acid profile and minimal residual sugars, aligning well with consumer expectations for bread flavor and texture. These results underscore that certain alternative *S. cerevisiae* strains have the potential to match or exceed the performance of commercial baker’s yeast, offering opportunities to optimize bread quality and diversify industrial baking practices.

## 1. Introduction

The preparation of bread and beer through yeast fermentation is one of the oldest fermentation processes known [1]. Despite its ancient origins, the intricacies of the fermentation process and its impact on product quality are not fully understood [2]. Historically, baker’s yeast has received less attention compared to brewer’s and wine-making yeasts [3]. The baking process typically involves fermentation dominated by *Saccharomyces cerevisiae*, known as baker’s yeast [4]. An ideal baker’s yeast should ensure uniform dough leavening, produce desirable flavors, and tolerate a wide range of temperatures, pH levels, and sugar and salt concentrations [5]. Previous studies have demonstrated that bread composition and quality can vary significantly depending on the yeast strain used. For instance, beer yeast strains *S. cerevisiae* T-58 and S-23 were found to enhance dough and bread quality, producing bread with higher specific volume, superior structural properties, and improved shelf life compared to standard baker’s yeast [2]. Yeast strains with robust metabolic activity and stress resistance tend to perform better in terms of fermentation efficiency and bread quality, leading to enhanced product consistency and consumer satisfaction.

In the context of industrial baking, commercial baker’s yeast is widely used due to its consistent performance and reliable fermentation characteristics. However, there is a growing interest in identifying alternative yeast strains that can match or surpass the performance of commercial baker’s yeast in terms of bread quality. This interest is driven by the need for diversification in yeast strain usage to cater to specific consumer preferences and to enhance the resilience of production processes against potential supply chain disruptions. Although there have been investigations on alternative yeast strains, the majority of these studies focus on non-*Saccharomyces* species [6,7]. It remains crucial to find *S. cerevisiae* strains with baking potential because *S. cerevisiae* is inherently well suited for bread fermentation due to its efficiency in CO_2_ production, which is essential for dough leavening, and its proven ability to produce desirable flavors and textures in the final bread product. Furthermore, *S. cerevisiae* has a well-documented metabolic versatility and stress resistance, making it a robust candidate for various fermentation conditions encountered in baking [8].

This study aims to evaluate a range of yeast strains, including those used for brewing ales, lagers, meads, and wines, and compare their impact on various bread quality parameters. By employing a comprehensive analytical approach, we provide a quantitative metric for assessing the overall similarity between each tested strain and the commercial baker’s yeast and systematically identify yeast strains that produce bread with quality attributes closely matching those of the commercial strain. This approach facilitates the selection of optimal yeast strains and underscores the critical role of yeast strain selection in influencing baking performance and bread quality attributes.

## 2. Materials and Methods

### 2.1. Ingredients and Recipe for Bread Making

The primary ingredients used in this study included high-gluten wheat flour (Gold Dragon Fish, Yihai Kerry, Shanghai, China) with 20% protein, 3% fat, and 24% carbohydrates. Dry yeast was purchased from Angel Yeast (Yichang, Hubei, China), and Anchor butter was purchased from Fonterra (Auckland, New Zealand). Salt and sugar were purchased online from suppliers in Beijing, China. Yeast strains were sourced from the culture collection of the Food Microbiology Laboratory at Jiangnan University (Wuxi, Jiangsu, China). The loaf recipe included 270 g of high-protein wheat flour, 162 g of water, 30 g of sugar, 3 g of salt, 13.5 g of butter, and 5 × 10^10^ CFU/mL of yeast.

### 2.2. Determination of CFU/mL in 1 OD Unit of Yeast Strains

To determine the colony-forming units per milliliter (CFU/mL) in 1 OD unit of the yeast strains, 1 g of yeast powder was inoculated into yeast extract peptone dextrose (YPD) medium (Oxoid, Basingstoke, UK) and incubated overnight. Next, 5 µL of the culture was streaked on YPD agar plates to isolate single colonies, which were incubated at 30 °C for 48 h. Isolated colonies were then picked, inoculated into YPD broth, grown to mid-log phase, mixed with sterile glycerol to a final concentration of 15%, and stored at −80 °C. For each strain, a colony from the glycerol stock was cultivated in YPD medium at 30 °C with shaking at 2795 g using an orbital shaker (Thermo Fisher Scientific, Waltham, MA, USA) until the culture reached the exponential phase. The optical density (OD) of the culture was measured at 600 nm (OD_600_) using a spectrophotometer (Bio-Rad, Hercules, CA, USA). Serial dilutions were prepared from the culture at 1 OD_600,_ and 100 µL from each dilution was spread onto YPD agar plates in triplicate. After incubating at 30 °C for 48 h, colonies were counted on plates containing 30–300 colonies, and the average count was used to calculate the CFU/mL.

### 2.3. Loaf Production

A quick fermentation method was utilized for bread production. High-gluten wheat flour, water, sugar, salt, and yeast were mixed using a stand mixer (KitchenAid, St. Joseph, MI, USA) at low speed (approximately 0.02 g) for 3 min, forming a shaggy dough with no dry flour. The dough was then mixed at high speed (approximately 0.18 g) for 5 min to facilitate the initial formation of the gluten network. Butter was then added to the dough, followed by mixing at low speed for 2 min and high speed for another 5 min to ensure full gluten development. The dough was then subjected to primary fermentation in a proofing chamber (Memmert, Schwabach, Germany) at 30 °C and 85% relative humidity for 1.5 h. After primary fermentation, the dough was divided into 150 g portions and flattened. These portions rested for 20 min, were then rolled, placed into baking molds, and returned to the proofing chamber for secondary fermentation at 30 °C and 85% relative humidity for an additional 1.5 h. Finally, the dough was baked in an oven (Rational, Landsberg am Lech, Germany) preheated to 170 °C and baked for 40 min. After baking, the bread was cooled to room temperature, sliced, and either stored at 4 °C or kept at room temperature depending on the requirements of specific experiments.

### 2.4. Measurement of Loaf Parameters

#### 2.4.1. Determination of Loaf Specific Volume

The weight of the bread was first measured using a precision scale (Mettler Toledo, Greifensee, Switzerland). The volume of the bread was determined using the rapeseed displacement method as described by Mudgil et al. [9]. A 2000 mL beaker was initially filled to the brim with rapeseeds, leveled at the mouth to establish a baseline volume. Some rapeseeds were then removed to make space for the loaf. The loaf sample was placed inside the beaker, and the removed rapeseeds were gradually added back until they fully covered the loaf. The volume of the loaf was determined by the amount of rapeseeds that could not be returned to the beaker. The specific volume of the bread (cm^3^/g) was calculated by dividing the volume (V) of the bread by its weight (M).

#### 2.4.2. Determination of Loaf pH

The pH of the bread was measured using a digital pH meter (Hanna Instruments, Woonsocket, RI, USA). Samples were prepared by homogenizing 10 g of loaf sample in 90 mL of distilled water. The mixture was stirred for 30 min to ensure a uniform suspension.

#### 2.4.3. Determination of Crumb Hardness

Bread hardness was measured using a texture analyzer (TA.XTplus, Stable Micro Systems, Godalming, Surrey, UK) equipped with a 36 mm cylindrical probe. The hardness test was performed on fresh bread (day 0) and on bread stored for 7 days at room temperature. Each loaf was sliced into 25 mm thick slices, and the compression test was conducted at a speed of 1.7 mm/s to a depth of 40% of the slice thickness. The maximum force required to compress the bread, recorded in grams (g), was taken as the hardness value. Each strain generated three samples, and measurements were obtained in triplicate for each sample and at each time point, resulting in a total of nine measurements.

#### 2.4.4. Determination of Residual Sugars and Organic Acids in Loaf

Bread samples (5 g) were homogenized with 50 mL of distilled water and centrifuged at 2795× *g* for 15 min. The supernatant was filtered through a 0.45 µm membrane filter (Millipore, Burlington, MA, USA). For residual sugars, the filtrate was analyzed using a high-performance liquid chromatography (HPLC) system (Agilent 1260 Infinity, Agilent Technologies, Santa Clara, CA, USA) equipped with a refractive index detector and a carbohydrate column (Hi-Plex Ca, stainless steel, Agilent Technologies, Santa Clara, CA, USA). The mobile phase consisted of acetonitrile and water (75:25, v/v) with a flow rate of 1.0 mL/min. Standard solutions of maltose, sucrose, fructose, and glucose (Sigma-Aldrich, St. Louis, MO, USA) were used for calibration. The concentration of each sugar was quantified by comparing the peak areas of the sample with those of the standards.

For organic acids, the same filtrate was analyzed on the HPLC system with an ultraviolet detector (VWD G1314F, Agilent Technologies, Santa Clara, CA, USA) set at 210 nm and a reverse-phase C18 column (ZORBAX Eclipse Plus C18, Agilent Technologies, Santa Clara, CA, USA). The mobile phase consisted of 0.01 M sulfuric acid, with a flow rate of 0.5 mL/min. Standard solutions of citric acid, malic acid, and acetic acid (Sigma-Aldrich, St. Louis, MO, USA) were used for calibration. The concentration of each acid was quantified by comparing the peak areas of the sample with those of the standards.

### 2.5. Statistical Analysis

#### 2.5.1. Normalization Using z-Scores

To evaluate the effect of different yeast strains on loaf quality, the mean for each parameter was calculated across three replicates for each strain. The analyzed parameters included specific volume, hardness on day 0, hardness on day 7, pH, residual sugars, and organic acids of the loaf samples. For comparative analysis between strains, all parameter values were normalized using z-scores. The normalization process involved calculating the mean (μ) and standard deviation (σ) for each parameter across all strains, and then transforming each parameter value (X) into a z-score using the following formula [10]:

$$z=\frac{X-\mu }{\sigma }$$

The mean (μ) for each parameter was computed as follows:

$$\mu =\frac{\sum_{i=1}^{n}{Parameter}_{i}}{n}$$

The standard deviation (σ) for each parameter was calculated using

$$\sigma =\sqrt{\frac{\sum_{i=1}^{n}{\left({Parameter}_{i}-\mu \right)}^{2}}{n}}$$

#### 2.5.2. Distance to ACY298

To identify the yeast strain most similar to the commercial strain (ACY298), the normalized values for all parameters were compared. First, the normalized value for ACY298 was calculated for each parameter:

$${\mathrm{Normalized}}_{\mathrm{commercial}}=\frac{{Parameter}_{commercial}-\mu }{\sigma }$$

Then, the absolute distance between each strain’s normalized value and the commercial strain’s normalized value was computed for each parameter:

Distance to commerical_i_ = ∣Normalized_i_ − Normalized_commercial_∣

#### 2.5.3. Overall Similarity Between Each Strain and ACY298

The Euclidean distance method was employed to measure the overall similarity between each yeast strain and ACY298 by considering multiple parameters simultaneously [11]. In this context, each of the yeast strain characteristics were treated as a point in a multi-dimensional space. Each dimension in this space represented one of the measured parameters.

The Euclidean distance (D) between two points *A* and *B* in an n-dimensional space is given by

$$D=\sqrt{\sum_{i=1}^{n}{\left(A_{i}-B_{i}\right)}^{\;2}}$$

where *A*_*i*_ and *B_i_* are the coordinates (values) of the points *A* and *B* in the *i*-th dimension. The points A and B correspond to the parameter values for each yeast strain and the commercial strain ACY298, respectively.

## 3. Results

The goal of this study was to evaluate a range of commercial yeast strains and compare their impact on various bread quality parameters to identify strains that produce bread with quality attributes closely matching those of commercial baker’s yeast. The parameters tested include loaf specific volume, pH, hardness, residual sugar, and organic acid. Table 1 provides detailed information on the yeast strains evaluated, sourced from suppliers including White Labs, Wyeast Laboratories, and Lallemand. These strains are used for brewing ales, lagers, meads, and wines, each with distinct fermentation properties. Additionally, ACY283, a laboratory *S. cerevisiae* strain derived from the well-characterized S288C background, was included [12]. The commercial baker’s yeast strain ACY 298, used as reference, is L’hirondelle Fresh Yeast from Lesaffre, known for its consistent performance and reliable fermentation characteristics.

### 3.1. Yeast Viability Assessment

The colony-forming units per milliliter (CFU/mL) per optical density (OD) unit (CFU/mL per OD unit) is a crucial measure for accurately determining the actual number of viable yeast cells in a culture, as the same OD does not necessarily correspond to the same cell count across different strains. This measure ensures that each strain is inoculated at an equivalent viable cell density in subsequent experiments, allowing for a more accurate comparison of their fermentation performance and impact on loaf quality. Variations in CFU/mL per OD unit among yeast strains can be attributed to genetic differences and cell size (Table 1). Genetic makeup affects growth rate, metabolic efficiency, and stress resistance, leading to higher CFU/mL per OD values in robust strains [13]. Smaller cell sizes, influenced by genetics, result in higher CFU counts per OD unit, as a given OD value represents more smaller cells compared to fewer larger cells [14]. Adaptation to the environment also plays a critical role. Strains well-adapted to specific growth conditions (nutrient availability, temperature, pH) exhibit higher viability and CFU/mL per OD values [15]. Enhanced stress response pathways and efficient nutrient uptake systems enable these strains to thrive, maintaining robustness and activity, which could in turn ensure efficient fermentation and improved bread quality.

### 3.2. Specific Volume

**Figure 1:**
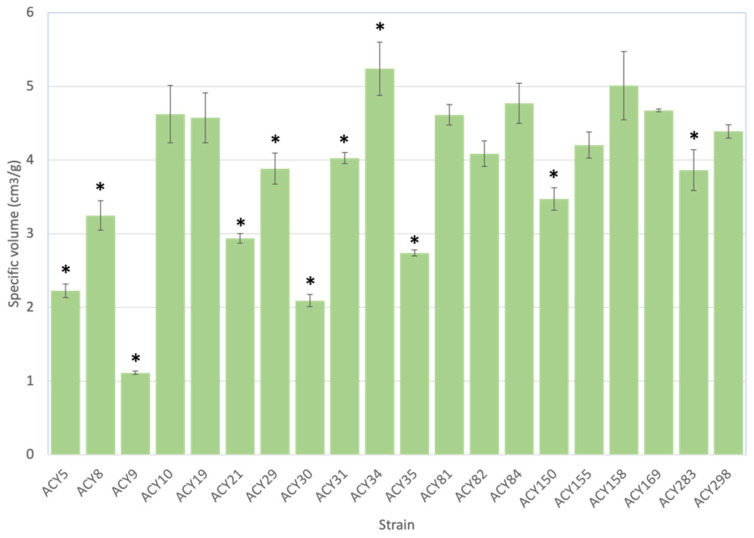
Specific volume of bread produced by different yeast strains. The specific volume (cm^3^/g) of bread produced by various yeast strains was measured and compared. Each bar represents the mean specific volume for a particular yeast strain, with error bars indicating the standard deviation (n = 3). The commercial strain ACY298 is used as a baseline for comparison. (*) indicates a significant difference between the commercial strain ACY298 and the tested strain (*p* < 0.05).

The specific volume of bread, a key quality parameter, was measured for different yeast strains (Figure 1). Significant variability in specific volume was observed among the strains, highlighting the influence of yeast strain selection on bread quality. Strains ACY10, ACY19, ACY81, ACY82, ACY84, ACY155, ACY158, and ACY169 produced bread with specific volumes comparable to the commercial strain ACY298, which had a specific volume of approximately 4.5 cm^3^/g. This suggests that these experimental strains exhibit fermentation performance similar to the commercial strain, making them viable alternatives for high-quality bread production due to their comparable gas production and retention capabilities during fermentation, which are crucial for achieving desirable bread volume and texture [16]. In contrast, strains such as ACY34 and ACY283 produced bread with significantly higher specific volumes, exceeding 5 cm^3^/g. This demonstrates superior performance in dough leavening due to enhanced gas production and retention. ACY34, achieving the highest specific volume, suggests potential for producing exceptionally light and airy bread. Conversely, strains like ACY9 and ACY30 resulted in significantly lower specific volumes, around 2 cm^3^/g, indicating less efficient fermentation activity, possibly due to lower cell viability or reduced gas production and retention. These strains might not be suitable for applications requiring high loaf volume, as they produce denser and less voluminous bread. Previous work has also observed that different strains can lead to differences in bread specific volume. For example, beer yeast strains used for bread making produced a range of specific volumes, with *S. cerevisiae* T-58 achieving the highest specific volume of 4.45 mL/g and *S. cerevisiae* US-05 producing a lower specific volume at 2.89 mL/g [2].

### 3.3. Crumb Hardness

**Figure 2:**
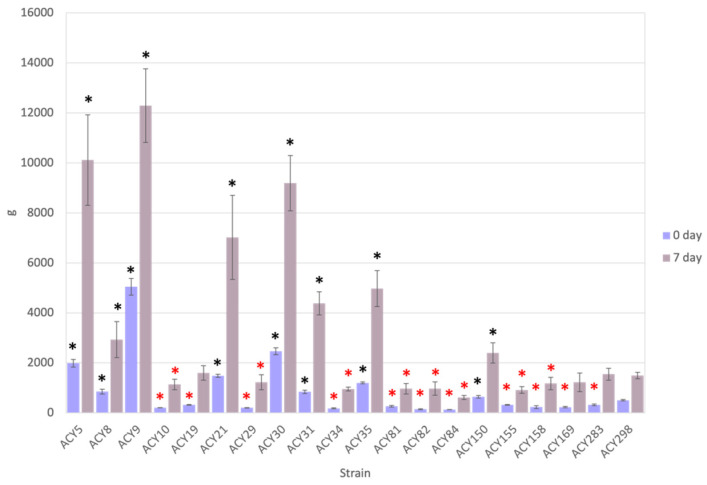
Bread hardness produced by yeast strains immediately after baking and after seven days of storage at 4 °C. Each bar represents the mean hardness for a particular yeast strain, with error bars indicating the standard deviation (n = 3). The commercial reference strain ACY298 is used as a baseline for comparison. Red asterisks indicate strains with significantly lower hardness compared to ACY298 (*p* < 0.05), while black asterisks indicate strains with significantly higher hardness compared to ACY298 (*p* < 0.05).

Bread hardness is a critical quality parameter that influences consumer preference and shelf life [17]. Softer bread is generally more desirable both immediately after baking and throughout its shelf life, as it indicates freshness and a pleasant eating experience. Crumb hardness was measured immediately after baking (day 0) and after seven days of storage (Figure 2). The results highlight significant variations in bread hardness among the tested strains, demonstrating the impact of yeast strain selection on bread texture and shelf life.

At day 0, bread hardness varied considerably among the yeast strains, with all strains showing significant differences from the commercial strain ACY298, which exhibited a moderate hardness value. Strains such as ACY5, ACY8, ACY21, ACY30, ACY31, ACY35, and ACY150 produced bread with significantly higher initial hardness values, suggesting a denser bread texture immediately after baking. This may be due to differences in dough structure and gas retention during fermentation. In contrast, strains like ACY9, ACY10, ACY19, ACY29, ACY34, ACY81, ACY82, ACY84, ACY155, ACY158, and ACY169 resulted in significantly lower initial hardness values, producing softer bread immediately after baking, which is often desirable. After seven days of storage, crumb hardness generally increased across all strains due to staling [18]. However, the extent of the increase varied significantly. Strains such as ACY5, ACY8, ACY9, ACY21, ACY30, ACY31, ACY35, and ACY150 continued to exhibit high hardness values, exceeding those of the commercial strain ACY298, suggesting that bread made with these strains stales more rapidly. Conversely, strains like ACY10, ACY29, ACY34, ACY81, ACY82, ACY84, ACY155, and ACY158 displayed significantly lower hardness values after seven days compared to ACY298, indicating better retention of bread softness and slower staling rates.

Notably, several strains, including ACY8, ACY31, ACY34, ACY81, ACY82, ACY84, ACY155, ACY158, and ACY169, produced bread with hardness values not significantly different from the commercial strain ACY298 at day 7. These strains demonstrated similar performance to ACY298 in maintaining lower hardness values over time, suggesting they might effectively replace the commercial strain in producing bread with comparable texture and shelf life. One recent work showed that crumb hardness and chewiness gradually decrease with increasing fermentation duration, with higher yeast concentrations resulting in softer bread [19]. Optimal yeast concentration (2–3%) and fermentation duration (90–105 min) were found to achieve desirable bread hardness. These parameters can be further optimized once the ideal alternative to the commercial strain is confirmed.

### 3.4. pH

**Figure 3:**
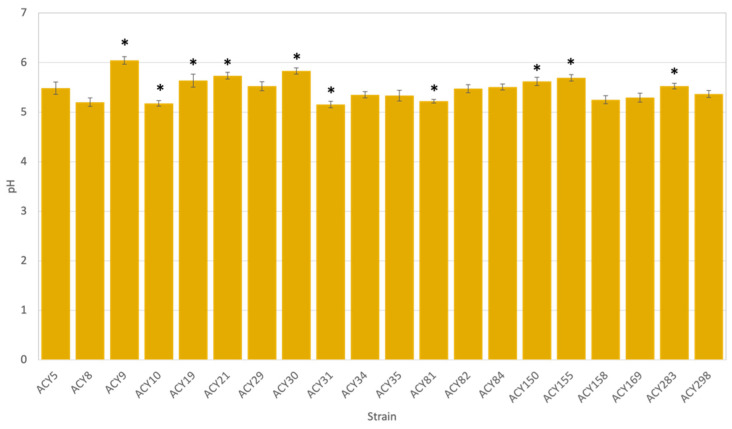
pH levels of bread samples. Each bar represents the mean pH for a particular yeast strain, with error bars indicating the standard deviation (n = 3). The commercial reference strain ACY298 is used as a baseline for comparison. (*) indicates a significant difference between the commercial strain ACY298 and the tested strain (*p* < 0.05).

Bread pH, typically ranging from 5.0 to 6.5, is a critical quality parameter influencing flavor, microbial stability, and overall quality [20]. The pH levels of bread produced by different yeast strains exhibit significant variability, underscoring the impact of yeast strain selection on bread acidity (Figure 3). The commercial strain ACY298 yielded bread with a pH level of approximately 5.5, reflecting moderate acidity. Strains such as ACY5, ACY8, ACY29, ACY34, ACY35, ACY82, ACY84, ACY158, and ACY169 produced bread with pH levels not significantly different from ACY298, suggesting these strains have fermentation profiles comparable to the commercial strain, making them suitable alternatives for producing bread with similar acidity level. Conversely, strains ACY9, ACY19, ACY21, ACY30, ACY150, ACY155, and ACY283 exhibited significantly higher pH values, indicating a less acidic environment. This lower acidity can be advantageous for producing bread with a milder flavor. In contrast, ACY10, ACY31, and ACY81 resulted in significantly lower pH values, indicating higher acidity. Higher acidity often contributes to a tangier flavor and enhanced microbial stability, which are desirable characteristics for certain bread types such as sourdough or rye bread [21].

### 3.5. Residual Sugars

**Figure 4:**
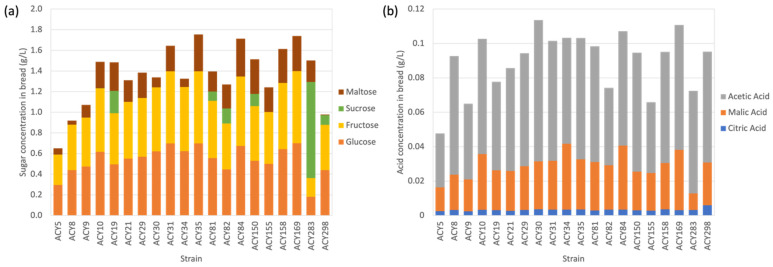
Residual sugar and organic acid profiles of bread: (**a**) shows the residual levels of glucose, fructose, sucrose, and maltose in bread for each yeast strain; (**b**) shows the residual levels of citric acid, malic acid, and acetic acid in bread for each yeast strain. The commercial reference strain ACY298 is used as a baseline for comparison.

Residual sugars after fermentation provide critical insights into the performance of different yeast strains in bread making. The residual levels of glucose, fructose, sucrose, and maltose in bread fermented by various yeast strains were measured to assess their efficiency in metabolizing these sugars (Figure 4a and Supplementary Figure S1). Most strains were efficient in fermenting glucose and fructose, though with some variability. Strains such as ACY31, ACY35, and ACY169 exhibited higher residual glucose and fructose levels (0.699 g/L and 0.700 g/L), indicating lower efficiency, and resulting in a sweeter bread. On the other hand, strain ACY283 had the lowest residual glucose and fructose level (0.181 g/L), suggesting higher fermentation efficiency and a less sweet taste, desirable for certain products. ACY298 demonstrated moderate efficiency in utilizing glucose and fructose, contributing to its versatility in producing a balanced flavor profile suitable for a wide range of baked goods.

The variability in sucrose utilization was pronounced. ACY283 exhibited the highest residual sucrose level (0.933 g/L), indicating inefficiency in sucrose metabolism. This higher residual sucrose could result in a sweeter final product, making ACY283 potentially suitable for sweet breads and pastries. Conversely, strains including ACY5, ACY8, ACY9, ACY21, ACY29, ACY30, ACY31, ACY34, ACY35, ACY84, ACY155, ACY158, and ACY169, showed no residual sucrose, suggesting complete or near-complete utilization. These strains are better suited for standard bread recipes where lower residual sweetness is preferred. ACY298 had a moderate residual sucrose level (0.093 g/L), indicating reasonable efficiency in sucrose utilization and making it suitable for a wide range of baked goods. Maltose utilization also varied significantly among the strains. ACY35 showed the highest residual maltose level (0.357 g/L), indicating lower efficiency. ACY19 and ACY10 also demonstrated relatively high residual maltose levels (0.276 g/L and 0.256 g/L). In contrast, ACY298 exhibited the lowest residual maltose level (0.007 g/L), indicating very efficient utilization, producing bread with a balanced flavor profile. Strains with higher residual maltose might be preferred in formulations requiring a slightly sweeter taste.

The selection of yeast strains based on their ability to metabolize residual sugars plays a crucial role in determining bread quality. Strains that leave higher levels of residual glucose, fructose, and sucrose tend to produce sweeter bread, whereas strains that efficiently utilize these sugars create bread with a more balanced flavor profile. Yeast activity, specifically the release and consumption of various sugars, also influences the sweetness and color of baked products. The predominant mono- and disaccharides found in white wheat flour include sucrose (2.16 mg/g of flour), fructose (0.91 mg/g of flour), maltose (0.53 mg/g of flour), and glucose (0.54 mg/g of flour) [22]. When yeast is present, sucrose does not directly contribute to sweetness because it is quickly hydrolyzed by yeast invertase into glucose and fructose [23]. Glucose and fructose have average relative sweetness values of 0.7 and 1.5, respectively, compared to sucrose, which is used as a reference for measuring sweetness [24]. In addition to glucose and fructose, other sugars in the flour, such as maltose and raffinose, along with fermentation by-products like polyols, significantly affect the sweetness of the final product. Maltose and raffinose have a relative sweetness of 0.50 and 0.22, respectively, while polyols vary in sweetness from 0.58 (mannitol) and 1.12 (xylitol) [25]. Moreover, reducing sugars like glucose and fructose contribute to color formation during baking through Maillard and caramelization reactions.

### 3.6. Organic Acids

Organic acids in bread enhance shelf life by creating an optimal acidic environment that improves amylase activity and antimicrobial properties, while also improving sensory quality by contributing to flavor, elasticity, ductility, and specific volume [26]. The synthesis of organic acids during fermentation offers critical insights into the metabolic efficiency of different yeast strains used in bread making (Figure 4b and Supplementary Figure S1). Citric acid, malic acid, and acetic acid levels were measured to assess their production across different strains. Citric acid synthesis was relatively consistent, with ACY298 producing the highest concentration at 0.0059 g/L. This indicates that ACY298 is particularly effective at producing citric acid, which enhances the acidity and tartness of bread, contributing to its freshness and flavor—especially desirable in artisan or specialty breads. Malic acid production varied more significantly, with ACY34 and ACY84 showing higher concentrations at 0.0383 g/L and 0.0372 g/L, respectively, indicating a robust capacity for malic acid synthesis. In contrast, ACY283 produced the lowest level at 0.0096 g/L, suggesting either rapid metabolism or reduced efficiency in malic acid production. Malic acid contributes a smooth, tart flavor to bread, with higher levels adding complexity to the flavor profile and lower levels resulting in a more balanced taste [27]. Acetic acid production also showed variability, with ACY30 producing the highest level at 0.0820 g/L, while ACY155 produced the lowest at 0.0412 g/L. Acetic acid imparts a vinegar-like taste to bread, which is especially important in enhancing the sourdough profile, where a strong acidic flavor is often preferred.

One previous investigation into the effects of organic acids on bread making revealed that breads containing 0.1% acetic acid, 0.4% lactic acid, 0.3% citric acid, 0.3% malic acid, and 0.3% fumaric acid showed an increase in specific volume and a decrease in moisture content, pH value, and hardness compared with the control [28]. The rheological behavior of the dough indicated that organic acids promote yeast activity, enhancing gas production capability. However, they also weaken the gluten network, leading to a reduction in gas retention capability. Overall, the synthesis of organic acids by yeast strains during fermentation significantly influences both the flavor profile and the overall quality of the bread. Strains that produce higher levels of citric, malic, and acetic acids yield bread with a more pronounced acidic and tangy flavor, while those with lower production contribute to a more balanced taste.

### 3.7. Comparative Analysis of Yeast Strains on Bread Quality

**Figure 5:**
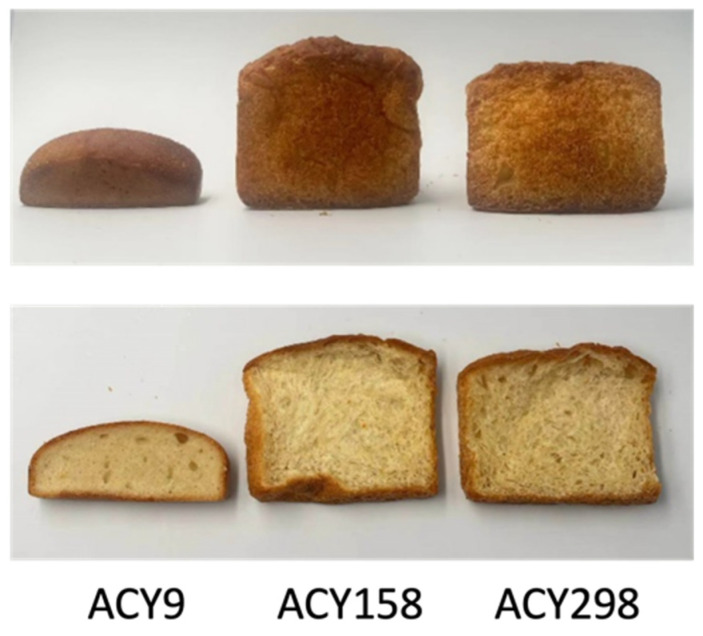
Comparison of bread made from the commercial yeast strain (ACY298) with bread produced using the most similar strain (ACY158) and the least similar strain (ACY9).

The comparison of various yeast strains to the commercial strain ACY298, using Euclidean distance as the metric, reveals significant differences in bread quality parameters such as specific volume, hardness, pH, residual sugars, and organic acids (Table 2). By normalizing all strains to ACY298 and calculating the Euclidean distances, the analysis identifies strains that closely match or diverge from ACY298, offering insights into their potential impact on bread quality. The validity of this method is demonstrated by using the most similar strain (ACY158), the least similar strain (ACY9), and ACY298 itself to make bread (Figure 5).

Strains ACY158, ACY169, and ACY81 emerged as the most similar to ACY298, with Euclidean distances of 0.895, 0.972, and 0.990, respectively. ACY158, the closest match, maintains a specific volume close to ACY298, along with low hardness values, suggesting it could produce bread with similar texture and flavor profile to ACY298. Both ACY169 and ACY81 show comparable texture and structure, with minor differences in pH and residual sugars, indicating consistent fermentation performance. These strains, due to their close resemblance to ACY298, are promising candidates for maintaining consistent bread quality in commercial baking. In contrast, strains like ACY9, ACY5, and ACY30, with Euclidean distances of 6.878, 5.353, and 4.290, respectively, show significant deviations from ACY298. ACY9, the least similar, exhibited a notably low specific volume, indicating a denser and less aerated bread structure. Its elevated hardness and pH values further suggest a firmer texture and potentially altered flavor, which may not be desirable for replicating the bread qualities of ACY298. Similarly, the higher pH and residual profiles of ACY5 and ACY30 indicate less efficient fermentation, potentially affecting flavor and shelf life, making these strains less suitable for achieving bread quality similar to ACY298.

Overall, the application of Euclidean distance as a quantitative measure in yeast strain selection highlights strains like ACY158, ACY169, and ACY81 as viable alternatives to ACY298 for producing consistent and desirable bread characteristics. Further research should focus on the practical application of these findings in commercial baking and explore the potential of these strains across various bread formulations.

## 4. Discussion

The results of our study highlight the critical role of yeast strain selection in shaping bread quality, providing valuable insights for both practical applications and future research in industrial baking. Strains like ACY158 demonstrated a strong potential for use as robust alternatives to commercial baker’s yeast, achieving similar outcomes in specific volume, crumb hardness, and acid profiles. These findings support the notion that the selection of yeast strains is a foundational element in optimizing bread quality, moving beyond its traditional secondary consideration. [29].

The superior performance of ACY158 compared to other alternative strains can be attributed to its efficient fermentation and balanced metabolic activity, which allowed it to produce gas, acids, and flavor compounds at levels comparable to ACY298. This raises questions about the specific enzymatic and regulatory networks that enable such high functional overlap despite differing strain origins. Future studies could focus on elucidating the genetic basis for these traits, which may include regulatory elements controlling glycolytic flux, organic acid pathways, and stress response mechanisms.

In contrast, the suboptimal performance of ACY9, characterized by a lower specific volume and greater hardness, underscores the complexity of yeast physiology. Strain-specific deficiencies in enzymatic activities, or cell wall dynamics could contribute to its poor fermentation outcomes. These findings align with previous studies demonstrating the sensitivity of bread quality to minor variations in yeast metabolism and stress tolerance [30].

The variability in organic acid synthesis across the strains presents intriguing opportunities for tailoring bread formulations. For instance, the balanced profiles of ACY158 are ideal for general-purpose bread, while higher acetic acid production in strains like ACY30 may cater to niche products such as sourdough or rye bread [31]. Organic acids not only impact flavor profiles but also contribute to microbial stability and texture during storage, potentially extending the shelf life of baked products. This highlights the importance of integrating acid profile assessments into yeast strain selection processes [32].

The application of Euclidean distance as a comparative tool offers a novel, quantitative approach to evaluating yeast strains. Unlike traditional single-parameter methods, this multidimensional analysis captures the holistic performance of each strain across multiple bread quality attributes. Expanding this methodology to include volatile aroma compounds or proteomic data could provide an even more comprehensive assessment, guiding the development of tailored strain profiles for specific applications.

From an industrial perspective, these findings open new avenues for strain engineering and adaptive evolution. By identifying the genetic and metabolic determinants of fermentation efficiency and resilience, targeted improvements can be made to enhance yeast performance under diverse baking conditions. This includes developing strains with improved stress tolerance, enhanced flavor production, and optimized gas retention capabilities. Such advancements would significantly expand the functional repertoire of baker’s yeast, enabling greater product diversity and consistency.

Beyond identifying alternative strains, our study underscores the intricate interplay between yeast metabolism and bread quality. Leveraging strains like ACY158 while addressing the metabolic bottlenecks in others opens new opportunities for innovation in bread production. By adopting a systematic approach to strain selection and engineering, the baking industry can achieve greater flexibility and alignment with evolving market demands.

## 5. Conclusions

The impact of yeast strain selection on bread quality, utilizing Euclidean distance as a robust tool for quantifying similarities across various parameters, has been demonstrated. ACY158, which closely matched the commercial strain ACY298, produced bread with comparable quality attributes, including specific volume, hardness, pH, and residual organic acid profiles. In contrast, ACY9, which exhibited the greatest deviation from ACY298, resulted in bread with distinct characteristics, such as reduced specific volume and altered texture. The validity of the Euclidean distance approach was confirmed through the successful production of bread using the most and least similar strains (ACY158 and ACY9). These findings suggest that certain alternative yeast strains have the potential to match or even exceed the performance of traditional commercial baker’s yeast, providing valuable opportunities for optimizing bread quality in industrial baking. Further research will focus on applying these findings across various bread formulations to better understand their commercial viability and alignment with consumer preferences.

## Figures and Tables

**Table 1:** Yeast strain information and relative CFU/mL per OD unit.

Strain Number	Strain Information	Number of CFU/mL in 1 OD Unit
ACY5	White Labs—WLP001 California Ale	5.33 × 10^6^ ± 3.21 × 10^5^ (*)
ACY8	Wyeast Laboratories—London ale	3.53 × 10^6^ ± 1.21 × 10^5^ (*)
ACY9	White Labs—WLP004 Irish Ale Yeast	6.23 × 10^6^ ± 8.33 × 10^5^ (*)
ACY10	White Labs—WLP300 Hefeweizen Ale Yeast	1.14 × 10^7^ ± 1.91 × 10^6^
ACY19	Wyeast Laboratories—Dry white/sparking	1.33 × 10^7^ ± 8.89 × 10^5^
ACY21	Wyeast Laboratories—Belgian style ale	7.23 × 10^6^ ± 1.27 × 10^6^ (*)
ACY29	Wyeast Laboratories—Sweet Mead	4.17 × 10^6^ ± 2.04 × 10^5^ (*)
ACY30	Wyeast Laboratories—Belgian high gravity	5.31 × 10^6^ ± 1.08 × 10^5^ (*)
ACY31	Wyeast Laboratories—Belgian abbey style ale	5.08 × 10^6^ ± 4.88 × 10^5^ (*)
ACY34	Wyeast Laboratories—Scottish ale	3.88 × 10^6^ ± 1.93 × 10^5^ (*)
ACY35	Wyeast Laboratories—Kolsch	3.88 × 10^6^ ± 1.95 × 10^5^ (*)
ACY81	Wyeast Laboratories—Belgian witbier	1.07 × 10^7^ ± 1.61 × 10^6^
ACY82	Wyeast Laboratories—American ale	8.87 × 10^6^ ± 4.04 × 10^5^
ACY84	Wyeast Laboratories—Irish ale	5.17 × 10^6^ ± 1.03 × 10^6^
ACY150	Lallemand—Lalvin 71B	1.18 × 10^7^ ± 2.03 × 10^6^ (*)
ACY155	Lallemand—Lalvin BM45	1.13 × 10^7^ ± 1.21 × 10^6^
ACY158	Lallemand—Lalvin ICV D254	7.00 × 10^6^ ± 6.56 × 10^5^
ACY169	Lallemand—Lalvin V1116	9.27 × 10^6^ ± 4.73 × 10^5^
ACY283	Lab strain S288C derivative	1.28 × 10^7^ ± 1.53 × 10^6^ (*)
ACY298	Lesaffre—L’hirondelle Fresh Yeast	1.02 × 10^7^ ± 1.73 × 10^6^

(*) indicates a significant difference between ACY298 and the tested strain (*p* < 0.05).

**Table 2:** Comparison of bread quality parameters and overall similarity of strains to ACY298.

Strain	Specific Volume	Hardness (Day 0)	Hardness (Day 7)	pH	Residual Sugars	Organic Acids	Euclidean Distance (D)
ACY5	1.941	1.279	2.479	0.495	2.876	2.930	5.353
ACY8	1.025	0.300	0.414	0.708	2.782	0.309	3.106
ACY9	2.940	3.926	3.104	2.873	1.292	1.923	6.878
ACY10	0.211	0.256	0.101	0.807	0.536	0.277	1.067
ACY19	0.167	0.163	0.030	1.146	0.023	1.179	1.661
ACY21	1.303	0.846	1.589	1.557	0.669	0.717	2.885
ACY29	0.454	0.262	0.075	0.665	0.929	0.209	1.276
ACY30	2.061	1.694	2.213	1.967	1.301	0.912	4.290
ACY31	0.325	0.291	0.830	0.906	0.537	0.210	1.426
ACY34	0.764	0.281	0.154	0.071	1.371	0.310	1.633
ACY35	1.481	0.602	1.001	0.142	0.600	0.307	2.008
ACY81	0.202	0.207	0.151	0.623	0.696	0.024	0.990
ACY82	0.270	0.311	0.149	0.453	1.073	1.385	1.862
ACY84	0.343	0.323	0.252	0.594	0.252	0.536	0.995
ACY150	0.823	0.120	0.261	1.076	0.125	0.196	1.404
ACY155	0.166	0.159	0.165	1.387	1.115	1.872	2.598
ACY158	0.559	0.235	0.091	0.495	0.390	0.166	0.895
ACY169	0.257	0.239	0.076	0.311	0.406	0.745	0.972
ACY283	0.472	0.161	0.016	0.679	0.074	1.489	1.712
ACY298	0	0	0	0	0	0	0

## Data Availability

The original contributions presented in this study are included in the article/Supplementary Material. Further inquiries can be directed to the corresponding authors.

## Supplementary Materials

The following supporting information can be downloaded at: https://www.mdpi.com/article/10.3390/microorganisms12122609/s1, Figure S1: High-Performance Liquid Chromatography (HPLC) Analysis of Residual Sugars and Organic Acids in Bread Produced by Various Yeast Strains.
