# Supplementary material for: Comparative Analysis of Bread Quality Using Yeast Strains from Alcoholic Beverage Production
Source: Microorganisms. 2024 Dec 17;12(12):2609. doi: 10.3390/microorganisms12122609 (PMC11676879; doi:10.3390/microorganisms12122609)
Supplement: Supplementary file 1 [file microorganisms-12-02609-s001.zip › microorganisms-3368737-supplementary.pptx]

## Slide 1
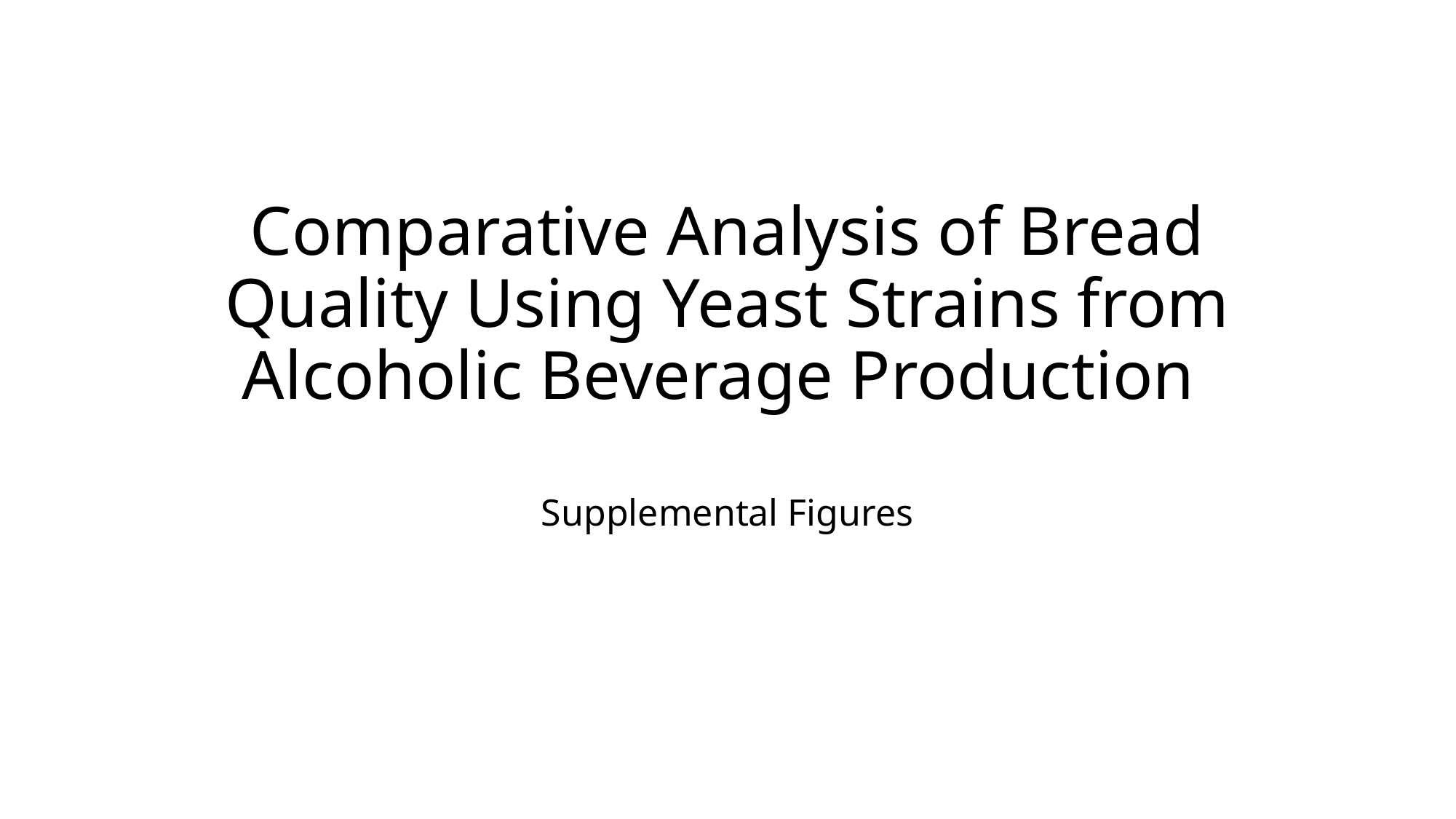

# Comparative Analysis of Bread Quality Using Yeast Strains from Alcoholic Beverage Production
Supplemental Figures

## Slide 2
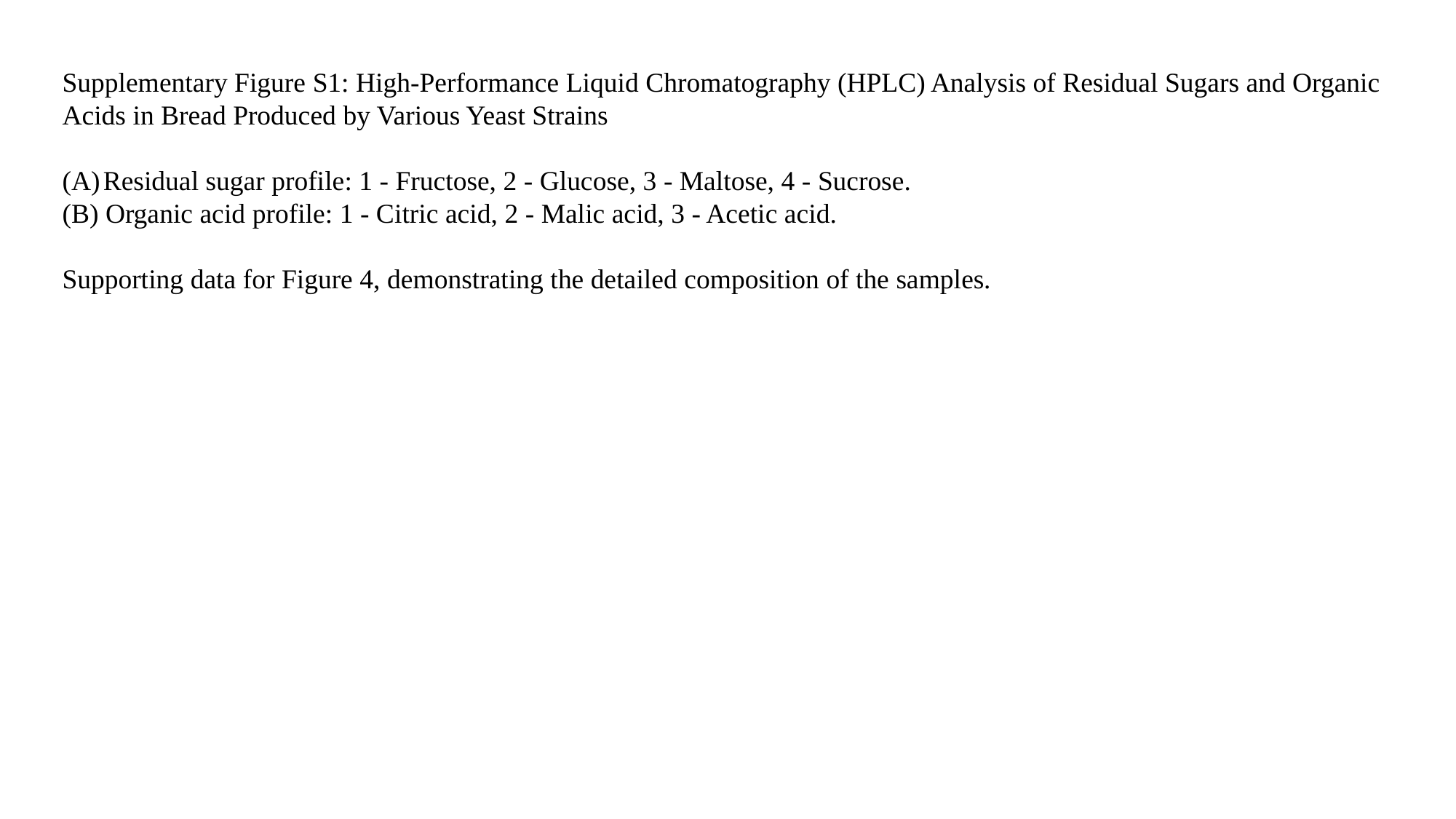

Supplementary Figure S1: High-Performance Liquid Chromatography (HPLC) Analysis of Residual Sugars and Organic Acids in Bread Produced by Various Yeast Strains
Residual sugar profile: 1 - Fructose, 2 - Glucose, 3 - Maltose, 4 - Sucrose.
(B) Organic acid profile: 1 - Citric acid, 2 - Malic acid, 3 - Acetic acid.
Supporting data for Figure 4, demonstrating the detailed composition of the samples.

## Slide 3
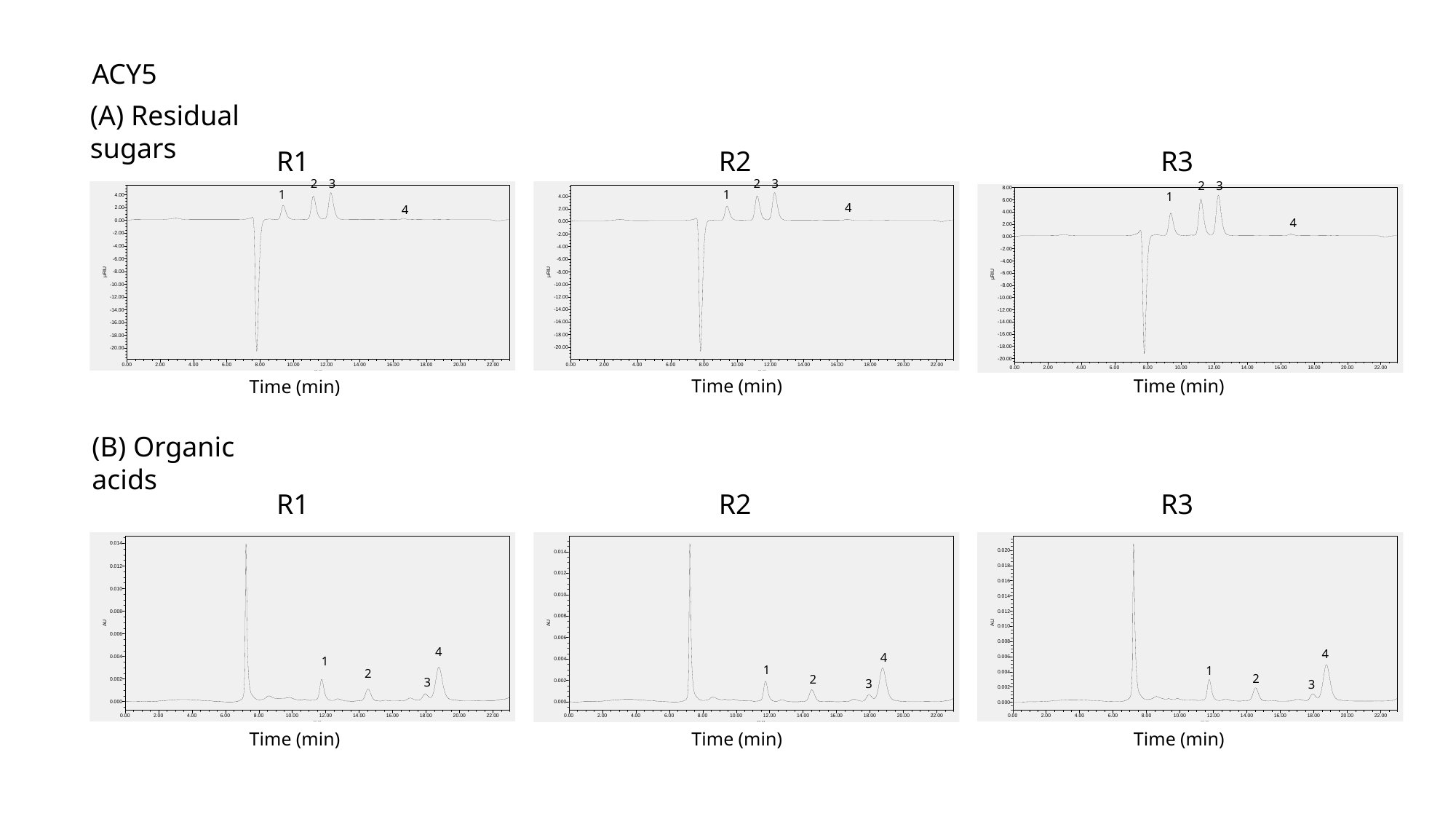

ACY5
(A) Residual sugars
R1
R2
R3
2
2
3
3
2
3
1
1
1
4
4
4
Time (min)
Time (min)
Time (min)
(B) Organic acids
R1
R2
R3
4
4
4
1
1
1
2
2
2
3
3
3
Time (min)
Time (min)
Time (min)

## Slide 4
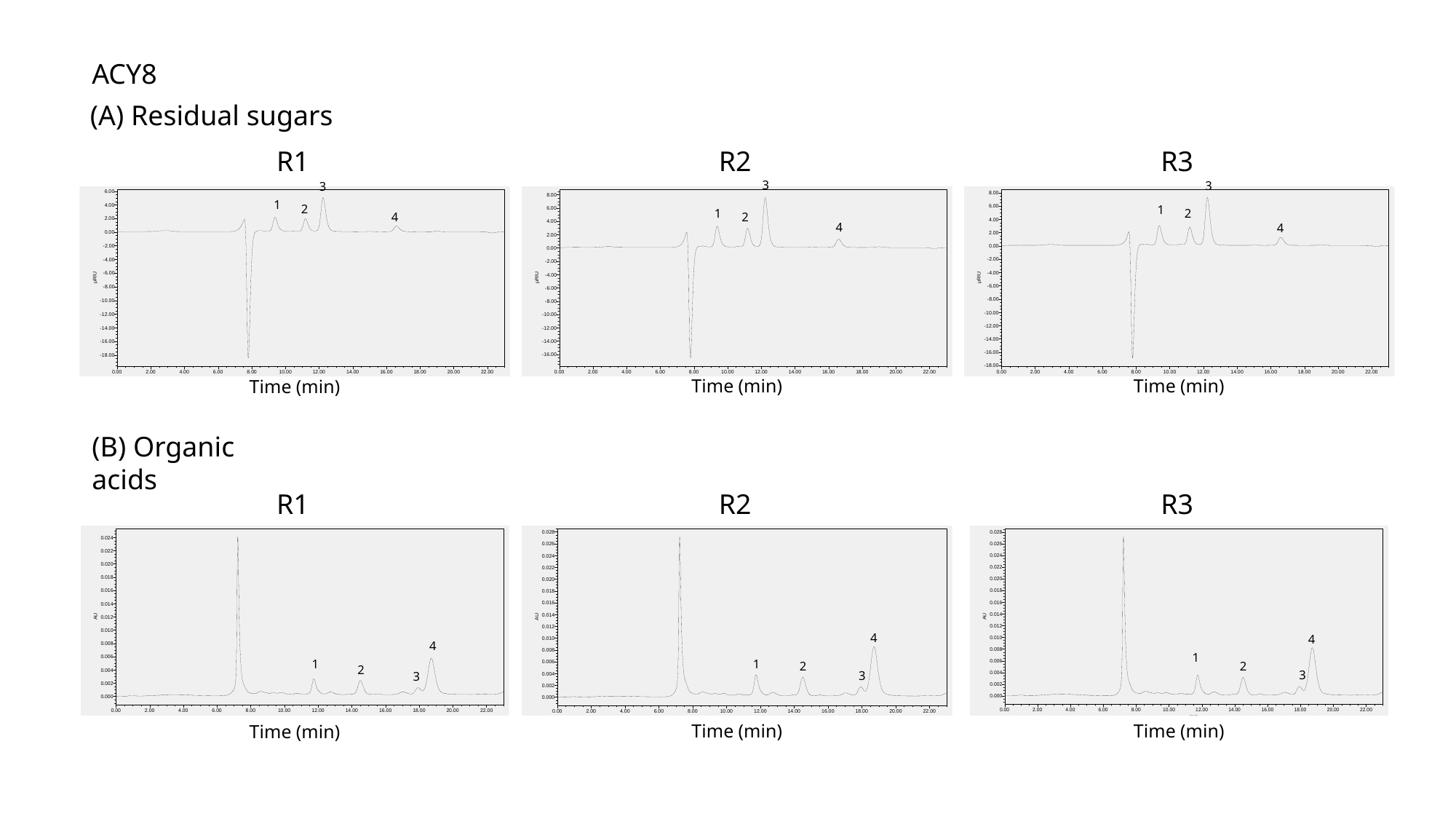

ACY8
(A) Residual sugars
R1
R2
R3
3
3
3
1
2
1
1
2
4
2
4
4
Time (min)
Time (min)
Time (min)
(B) Organic acids
R1
R2
R3
4
4
4
1
1
1
2
2
2
3
3
3
Time (min)
Time (min)
Time (min)

## Slide 5
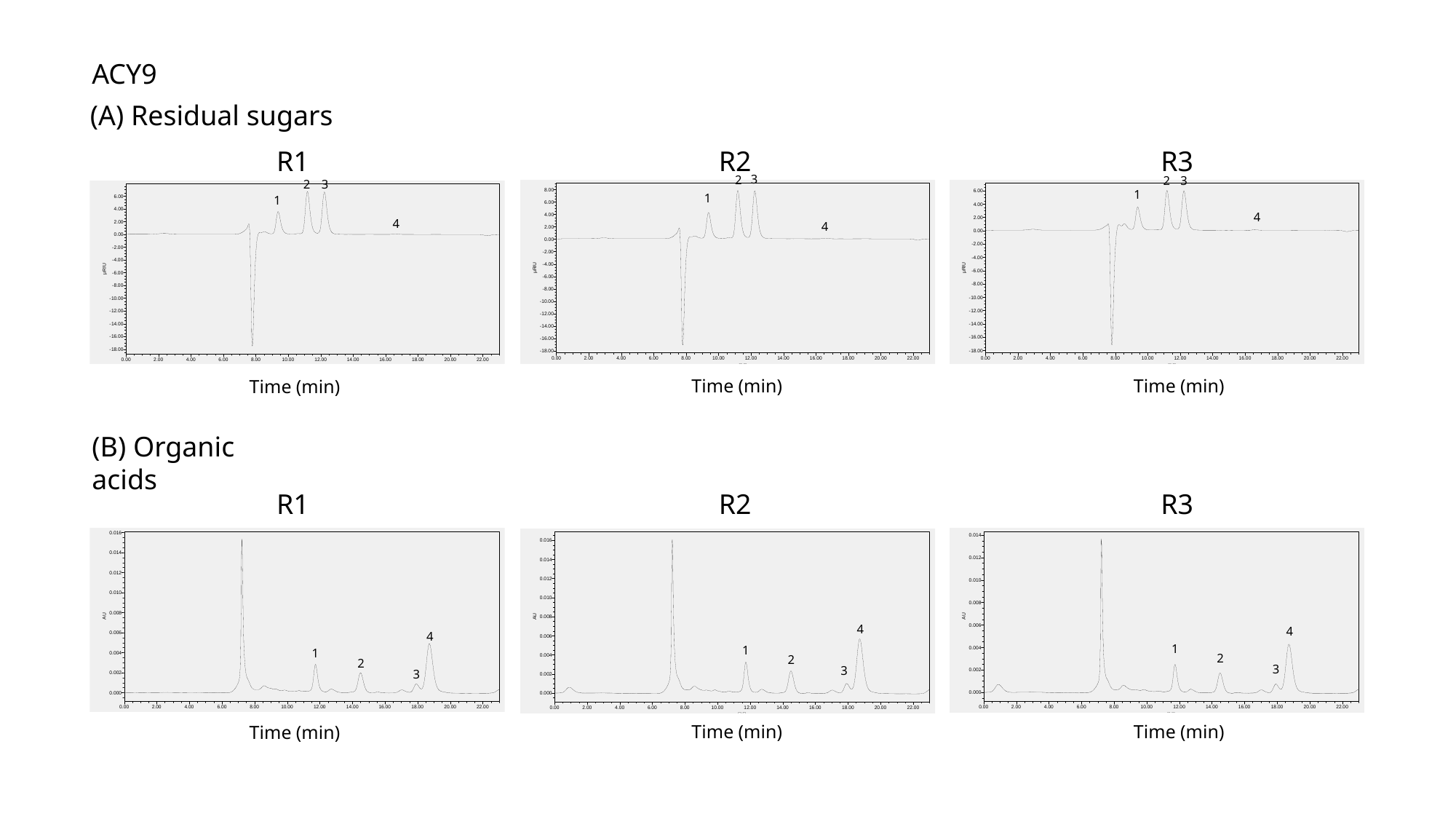

ACY9
(A) Residual sugars
R1
R2
R3
3
2
3
2
3
2
1
1
1
4
4
4
Time (min)
Time (min)
Time (min)
(B) Organic acids
R1
R2
R3
4
4
4
1
1
1
2
2
2
3
3
3
Time (min)
Time (min)
Time (min)

## Slide 6
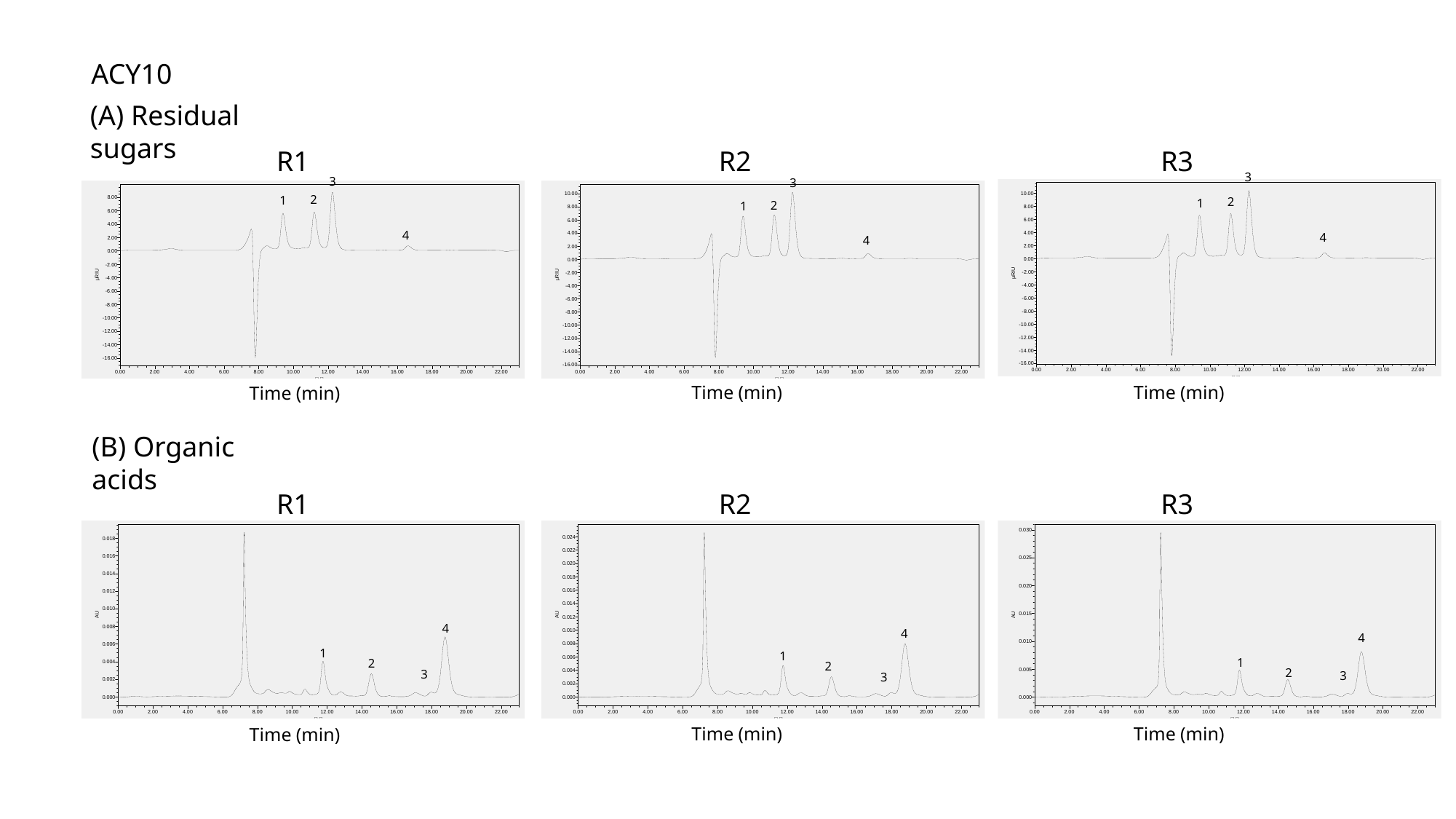

ACY10
(A) Residual sugars
R1
R2
R3
3
3
3
2
1
2
1
2
1
4
4
4
Time (min)
Time (min)
Time (min)
(B) Organic acids
R1
R2
R3
4
4
4
1
1
1
2
2
2
3
3
3
Time (min)
Time (min)
Time (min)

## Slide 7
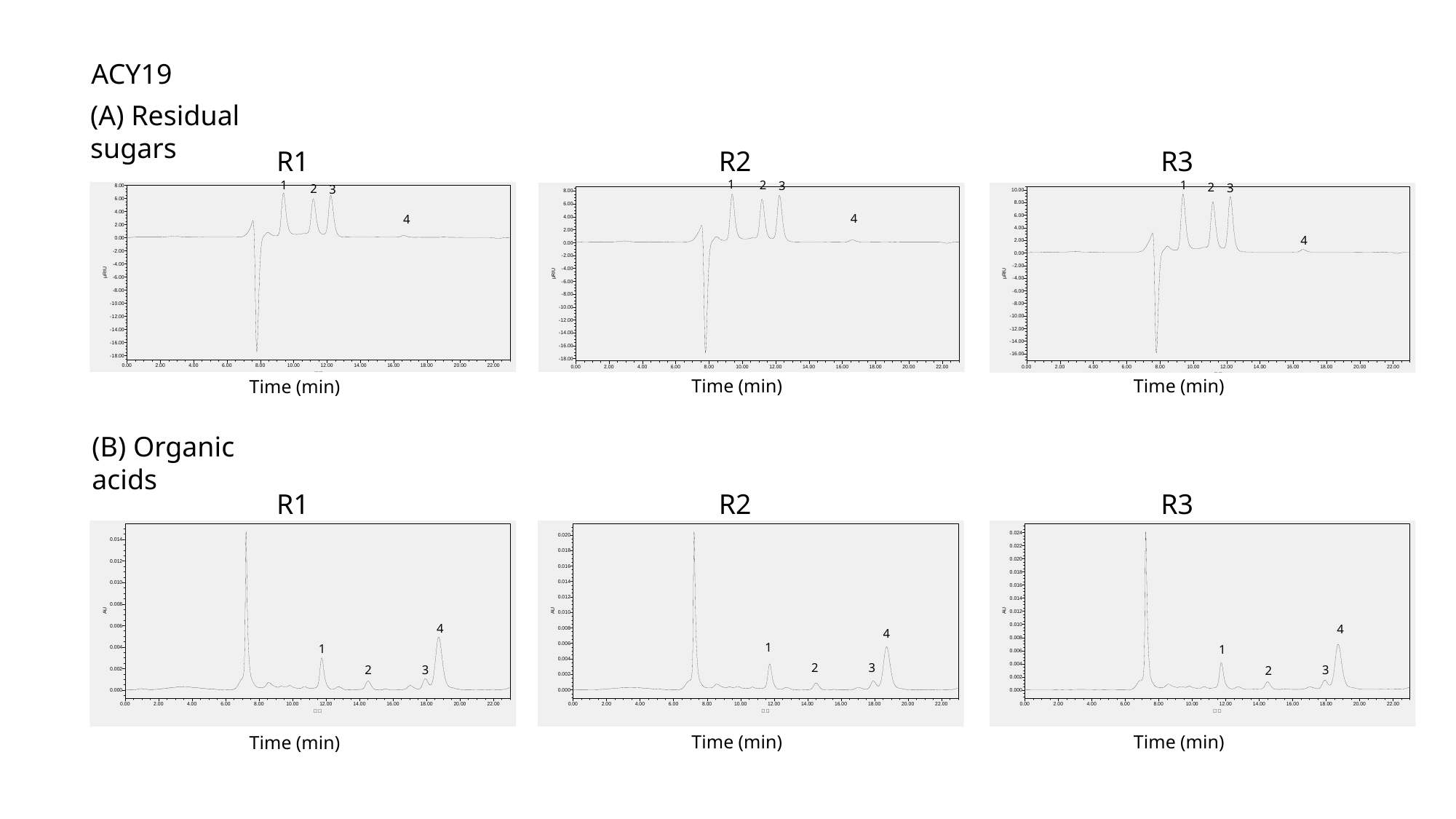

ACY19
(A) Residual sugars
R1
R2
R3
1
1
2
1
3
2
3
2
3
4
4
4
Time (min)
Time (min)
Time (min)
(B) Organic acids
R1
R2
R3
4
4
4
1
1
1
3
2
3
2
3
2
Time (min)
Time (min)
Time (min)

## Slide 8
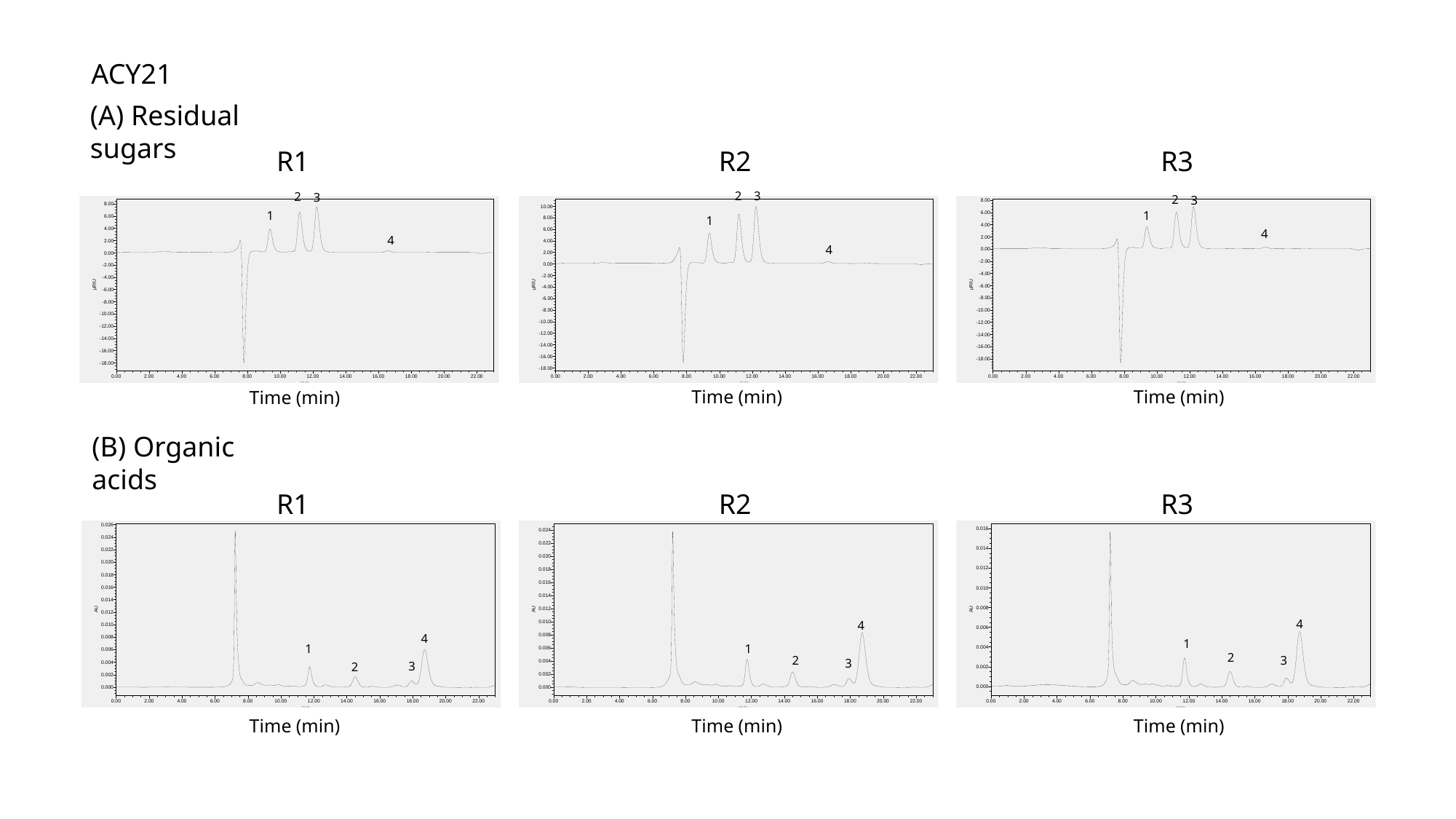

ACY21
(A) Residual sugars
R1
R2
R3
2
3
2
3
2
3
1
1
1
4
4
4
Time (min)
Time (min)
Time (min)
(B) Organic acids
R1
R2
R3
4
4
4
1
1
1
2
2
3
3
3
2
Time (min)
Time (min)
Time (min)

## Slide 9
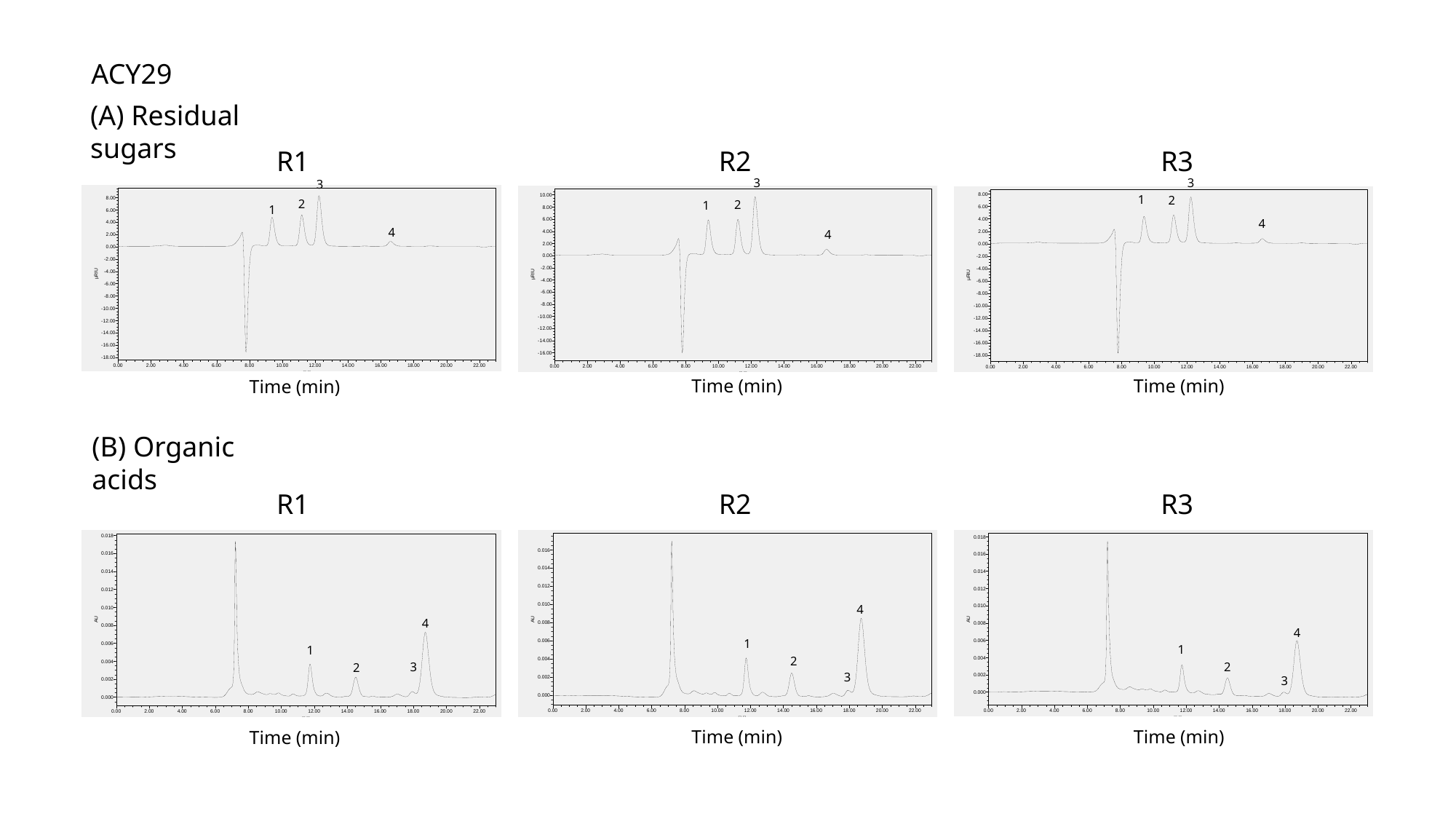

ACY29
(A) Residual sugars
R1
R2
R3
3
3
3
1
2
2
2
1
1
4
4
4
Time (min)
Time (min)
Time (min)
(B) Organic acids
R1
R2
R3
4
4
4
1
1
1
2
2
3
2
3
3
Time (min)
Time (min)
Time (min)

## Slide 10
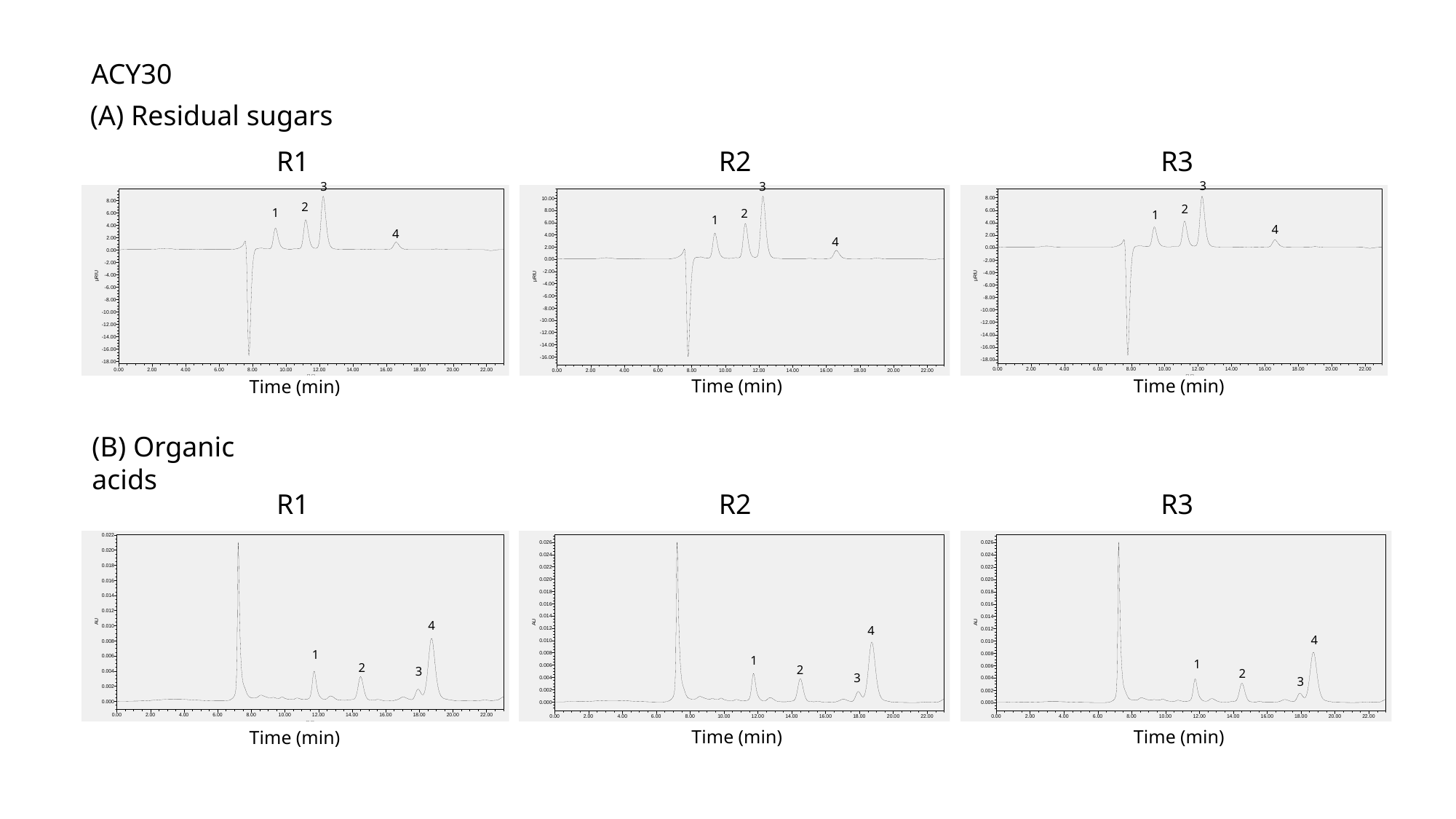

ACY30
(A) Residual sugars
R1
R2
R3
3
3
3
2
2
1
2
1
1
4
4
4
Time (min)
Time (min)
Time (min)
(B) Organic acids
R1
R2
R3
4
4
4
1
1
1
2
2
3
2
3
3
Time (min)
Time (min)
Time (min)

## Slide 11
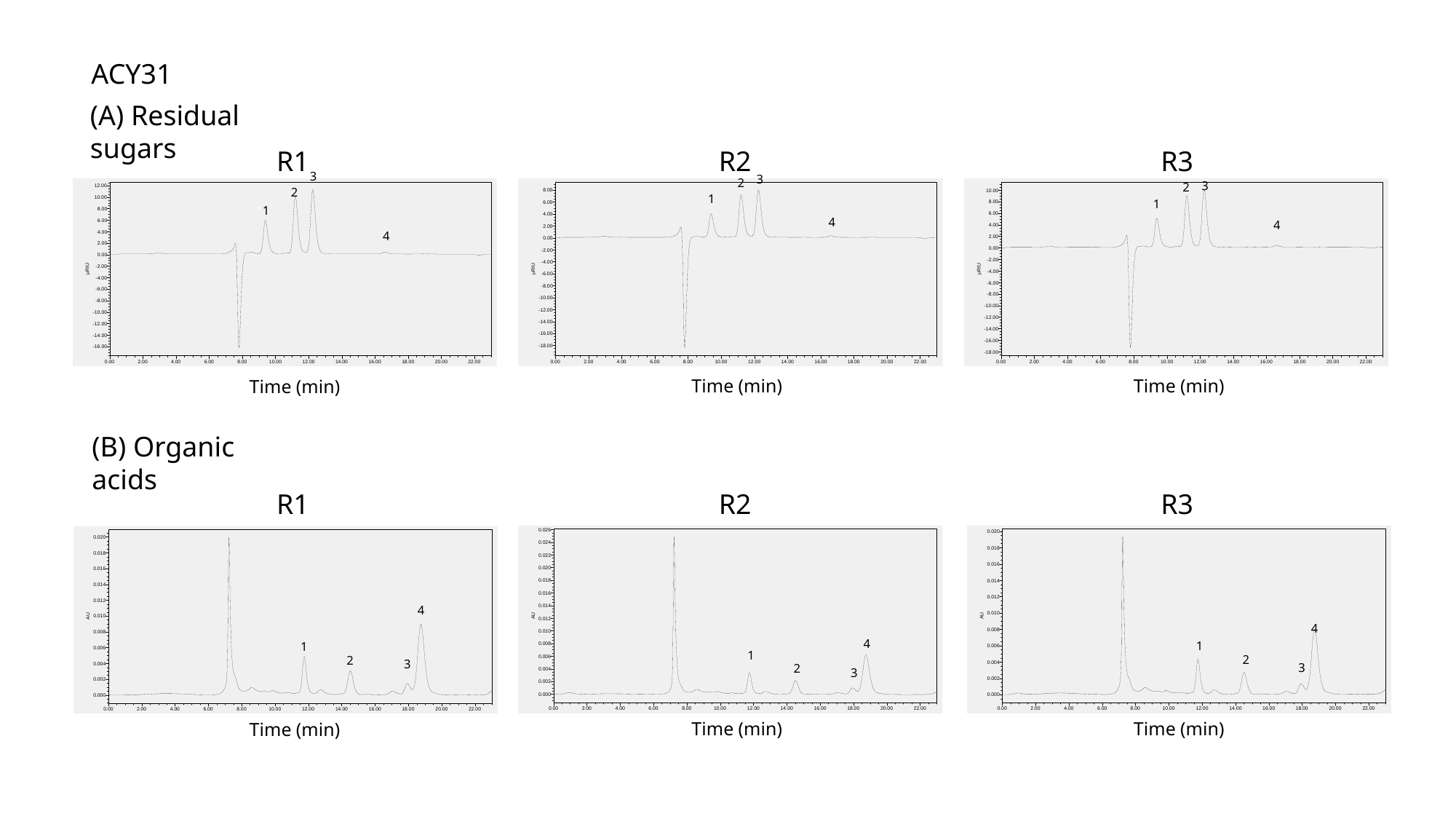

ACY31
(A) Residual sugars
R1
R2
R3
3
3
2
3
2
2
1
1
1
4
4
4
Time (min)
Time (min)
Time (min)
(B) Organic acids
R1
R2
R3
4
4
4
1
1
1
2
2
3
3
2
3
Time (min)
Time (min)
Time (min)

## Slide 12
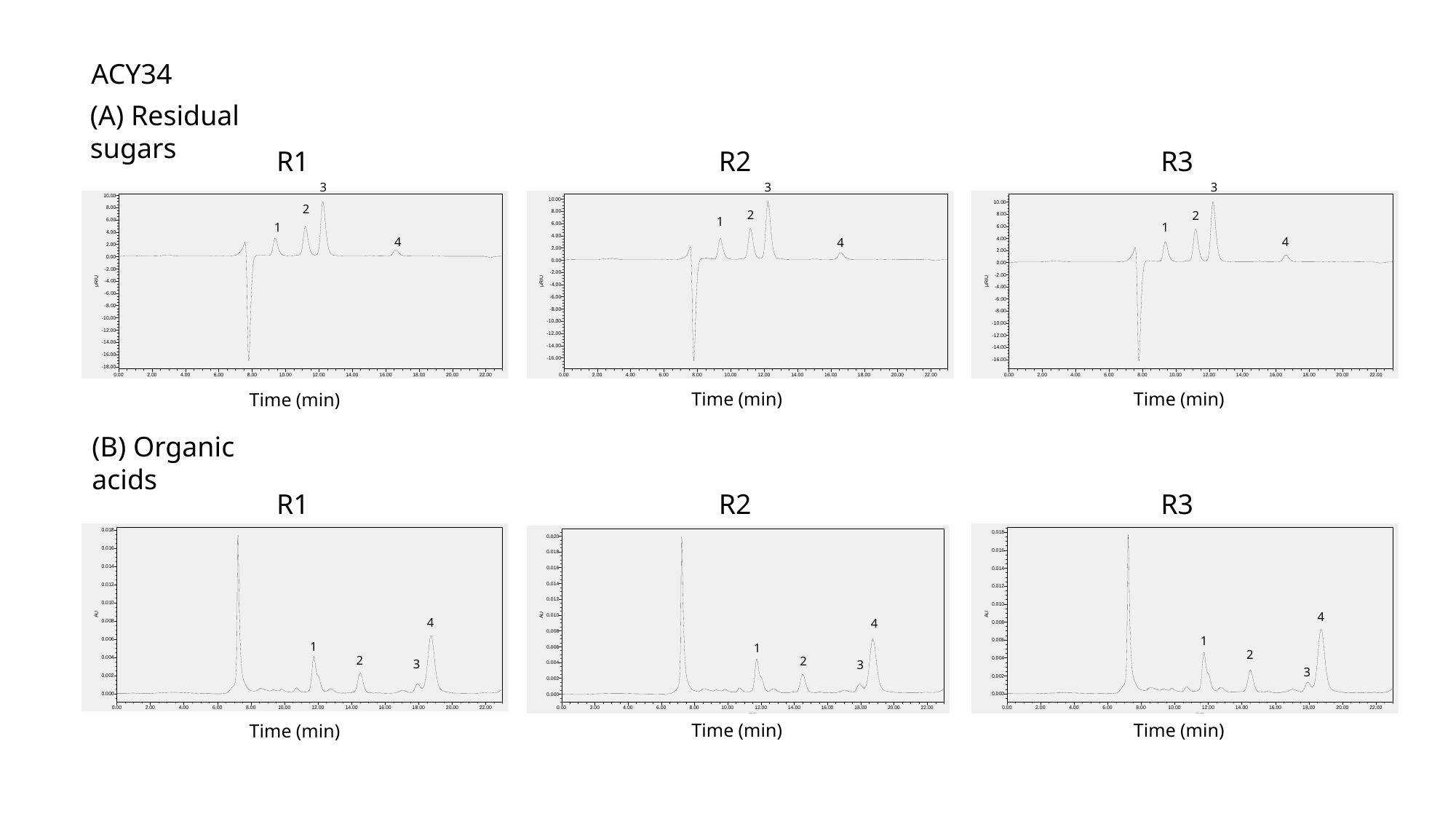

ACY34
(A) Residual sugars
R1
R2
R3
3
3
3
2
2
2
1
1
1
4
4
4
Time (min)
Time (min)
Time (min)
(B) Organic acids
R1
R2
R3
4
4
4
1
1
1
2
2
2
3
3
3
Time (min)
Time (min)
Time (min)

## Slide 13
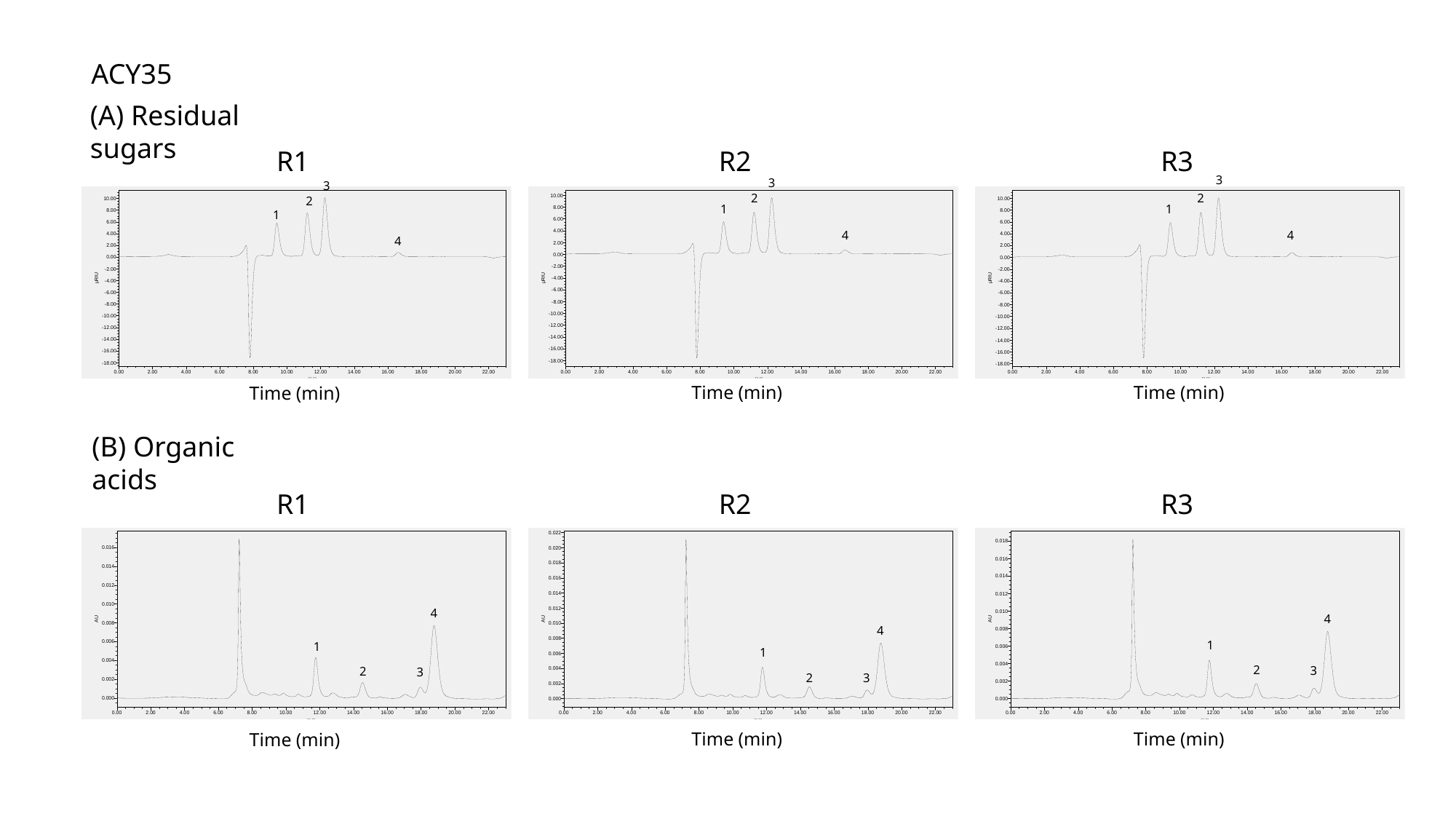

ACY35
(A) Residual sugars
R1
R2
R3
3
3
3
2
2
2
1
1
1
4
4
4
Time (min)
Time (min)
Time (min)
(B) Organic acids
R1
R2
R3
4
4
4
1
1
1
2
3
2
3
2
3
Time (min)
Time (min)
Time (min)

## Slide 14
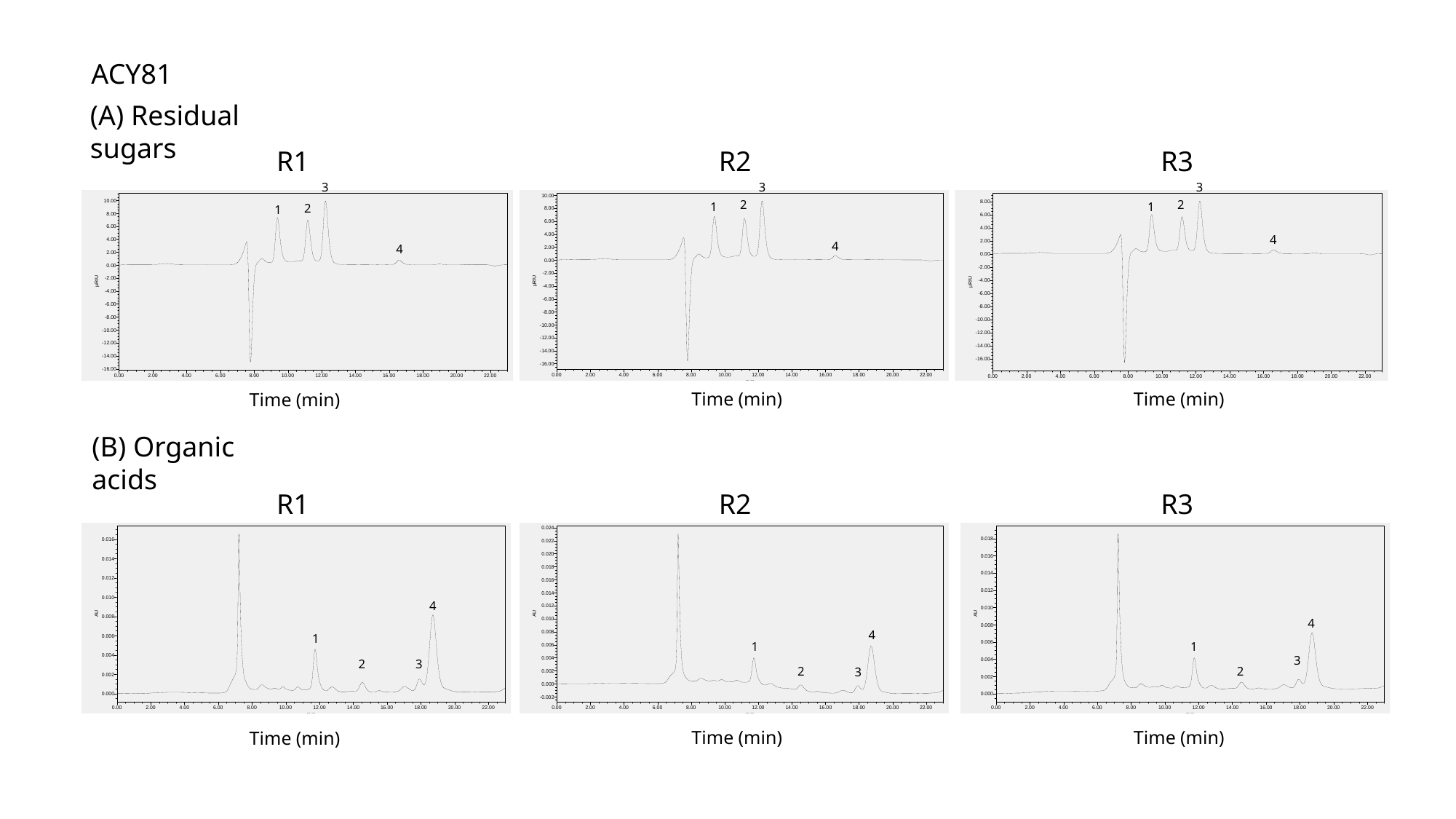

ACY81
(A) Residual sugars
R1
R2
R3
3
3
3
2
2
1
1
2
1
4
4
4
Time (min)
Time (min)
Time (min)
(B) Organic acids
R1
R2
R3
4
4
4
1
1
1
3
2
3
2
2
3
Time (min)
Time (min)
Time (min)

## Slide 15
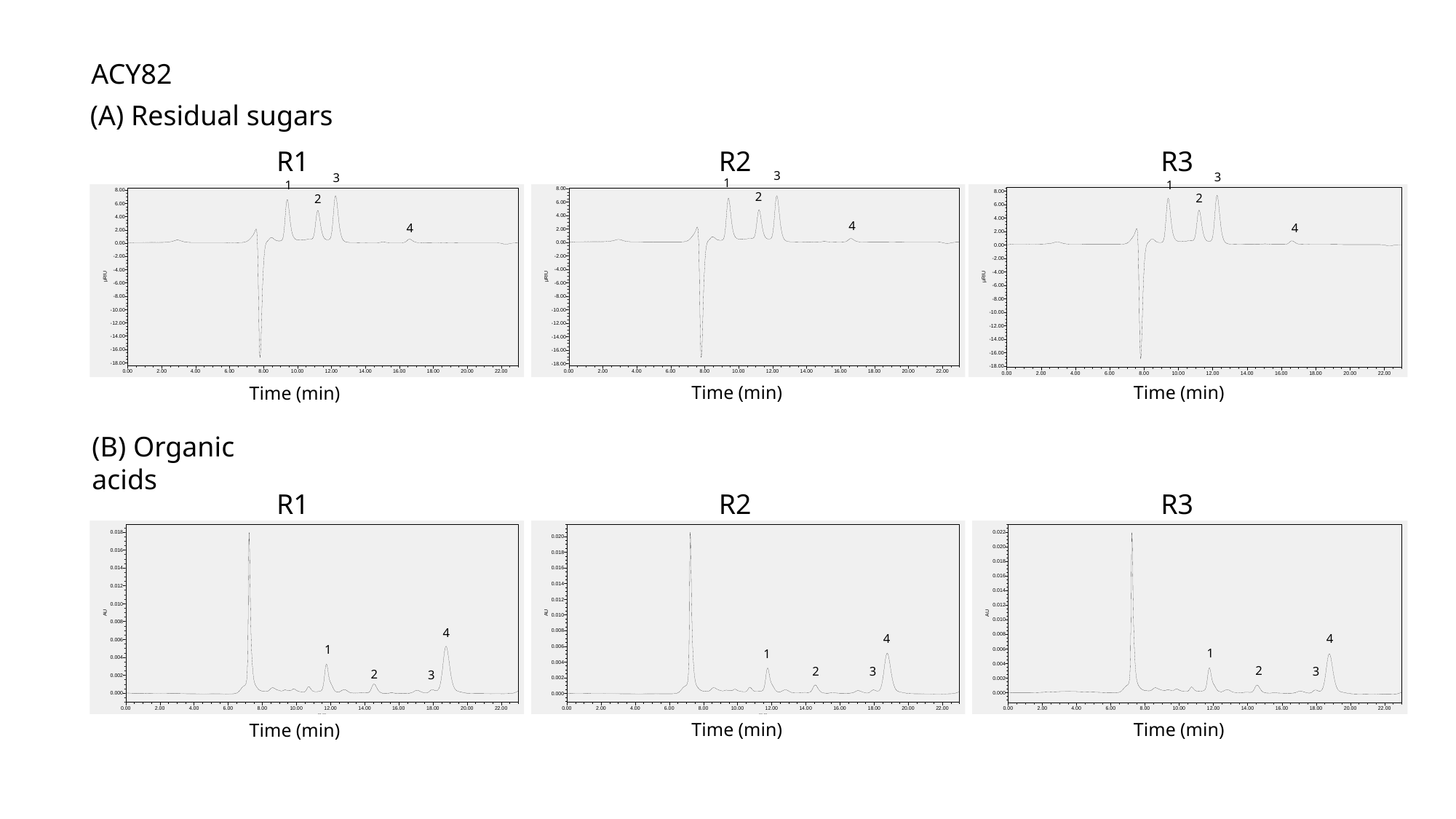

ACY82
(A) Residual sugars
R1
R2
R3
3
3
3
1
1
1
2
2
2
4
4
4
Time (min)
Time (min)
Time (min)
(B) Organic acids
R1
R2
R3
4
4
4
1
1
1
2
3
2
3
2
3
Time (min)
Time (min)
Time (min)

## Slide 16
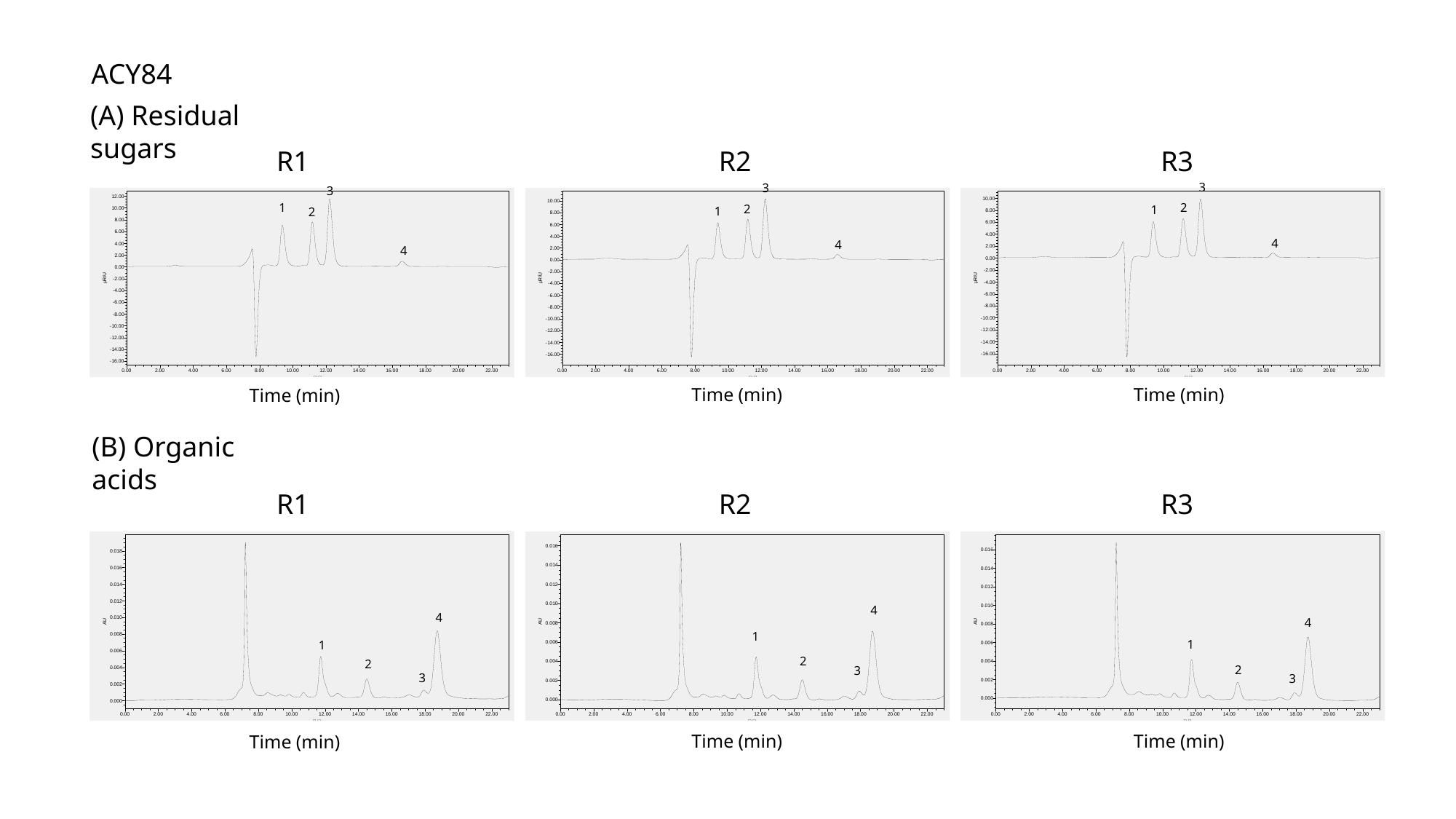

ACY84
(A) Residual sugars
R1
R2
R3
3
3
3
1
2
2
1
1
2
4
4
4
Time (min)
Time (min)
Time (min)
(B) Organic acids
R1
R2
R3
4
4
4
1
1
1
2
2
2
3
3
3
Time (min)
Time (min)
Time (min)

## Slide 17
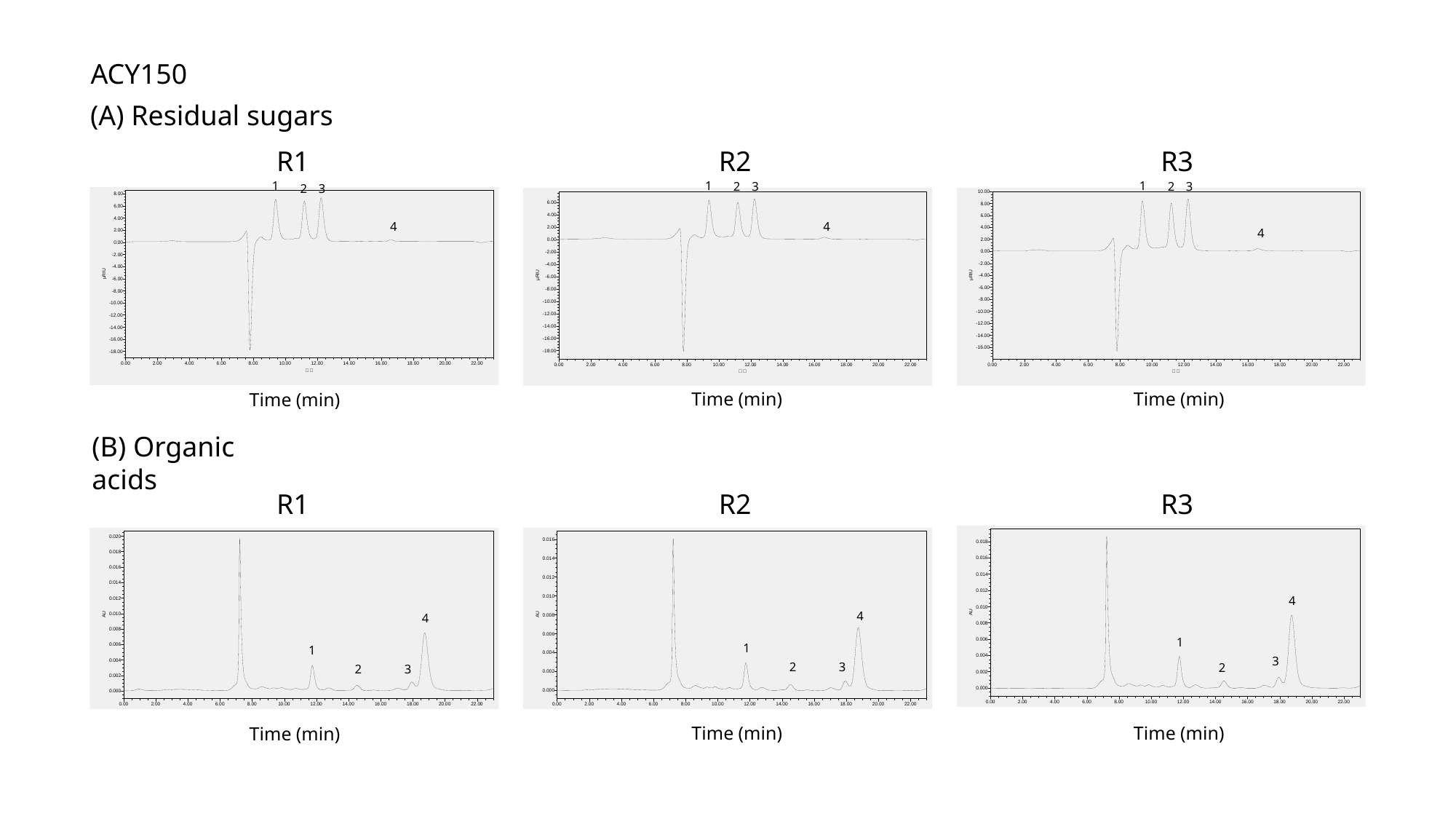

ACY150
(A) Residual sugars
R1
R2
R3
1
1
1
3
3
2
2
3
2
4
4
4
Time (min)
Time (min)
Time (min)
(B) Organic acids
R1
R2
R3
4
4
4
1
1
1
3
2
3
2
2
3
Time (min)
Time (min)
Time (min)

## Slide 18
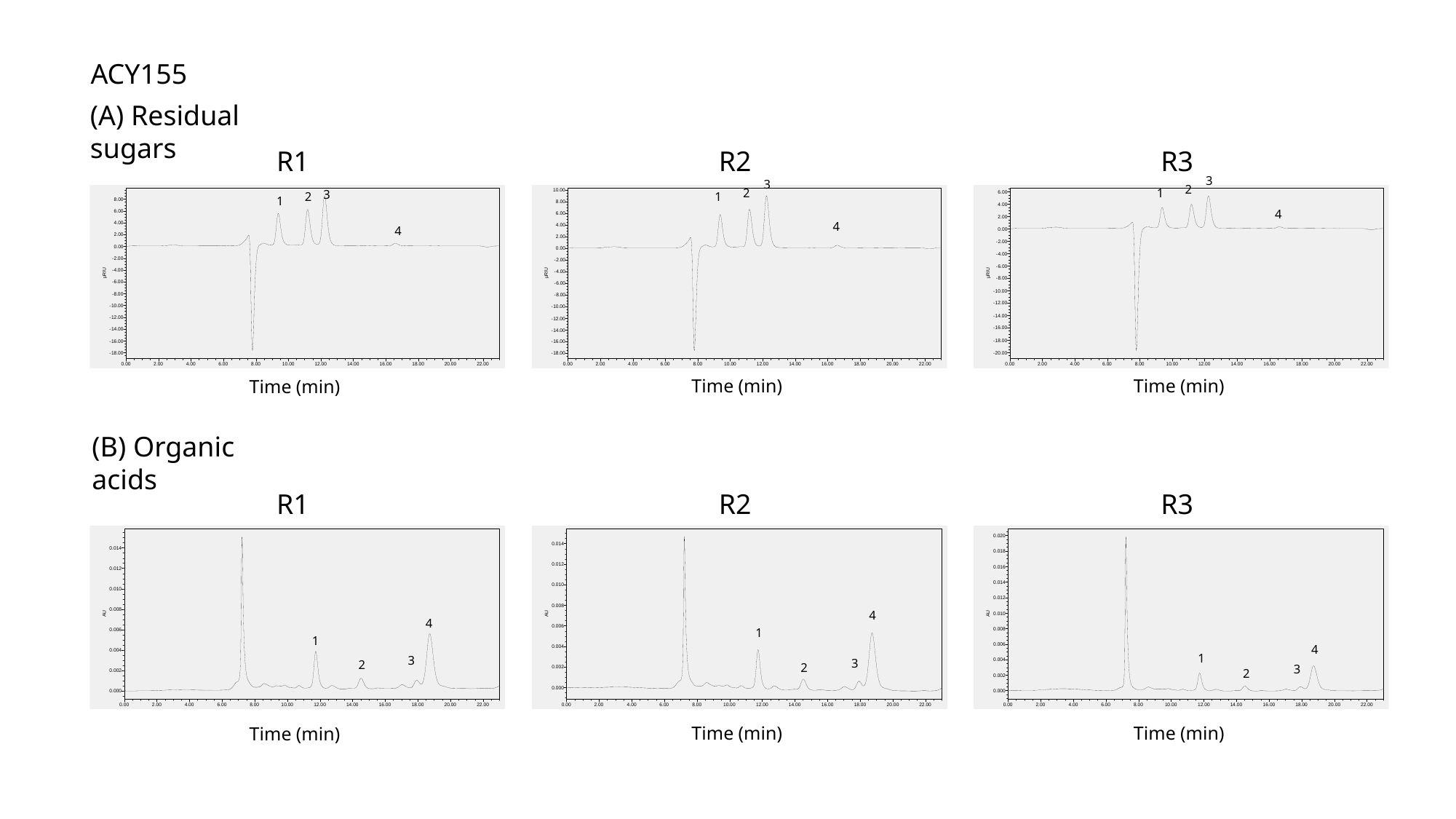

ACY155
(A) Residual sugars
R1
R2
R3
3
3
2
2
1
3
1
2
1
4
4
4
Time (min)
Time (min)
Time (min)
(B) Organic acids
R1
R2
R3
4
4
1
1
4
1
3
3
2
2
3
2
Time (min)
Time (min)
Time (min)

## Slide 19
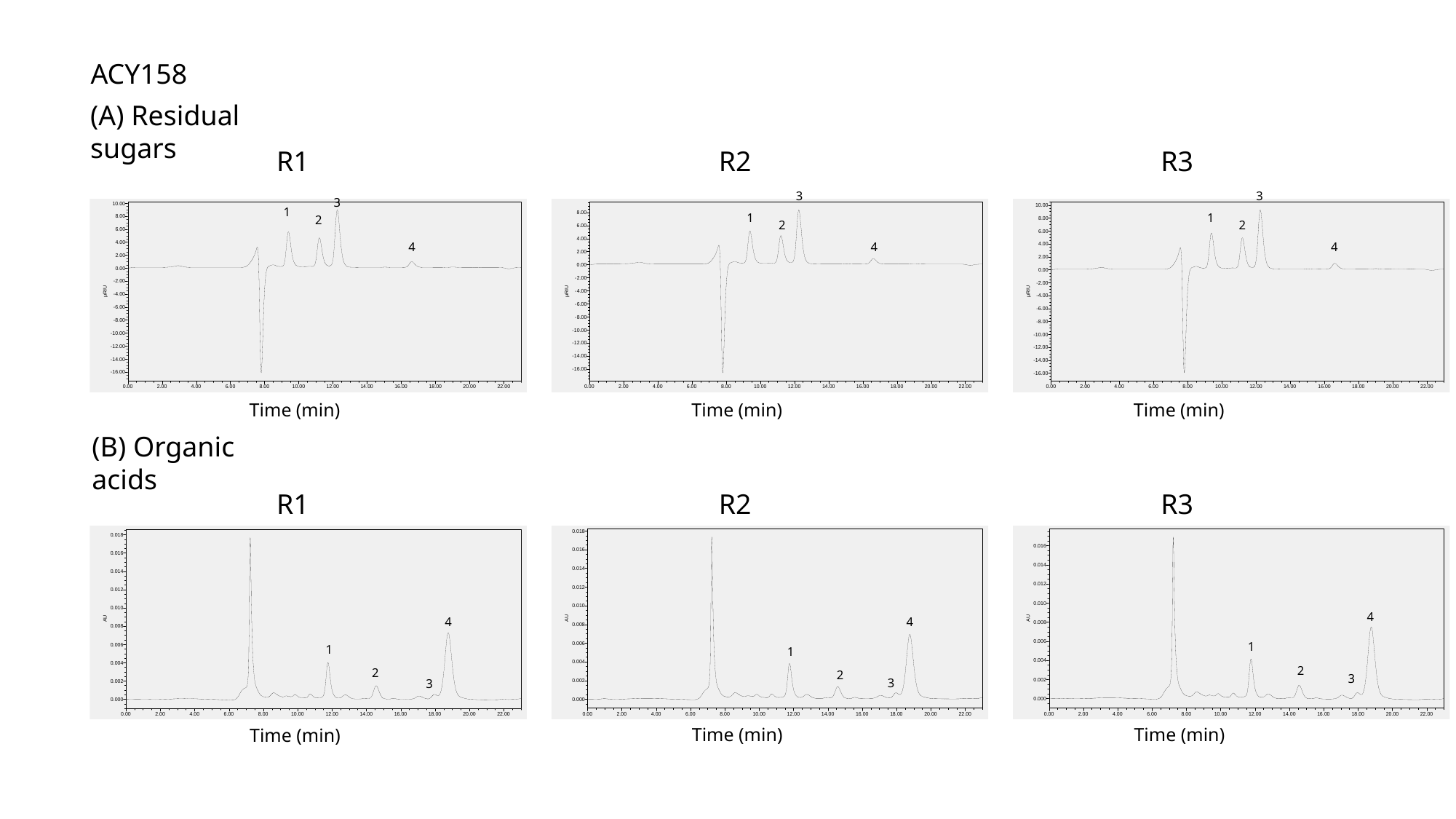

ACY158
(A) Residual sugars
R1
R2
R3
3
3
3
1
1
1
2
2
2
4
4
4
Time (min)
Time (min)
Time (min)
(B) Organic acids
R1
R2
R3
4
4
4
1
1
1
2
2
2
3
3
3
Time (min)
Time (min)
Time (min)

## Slide 20
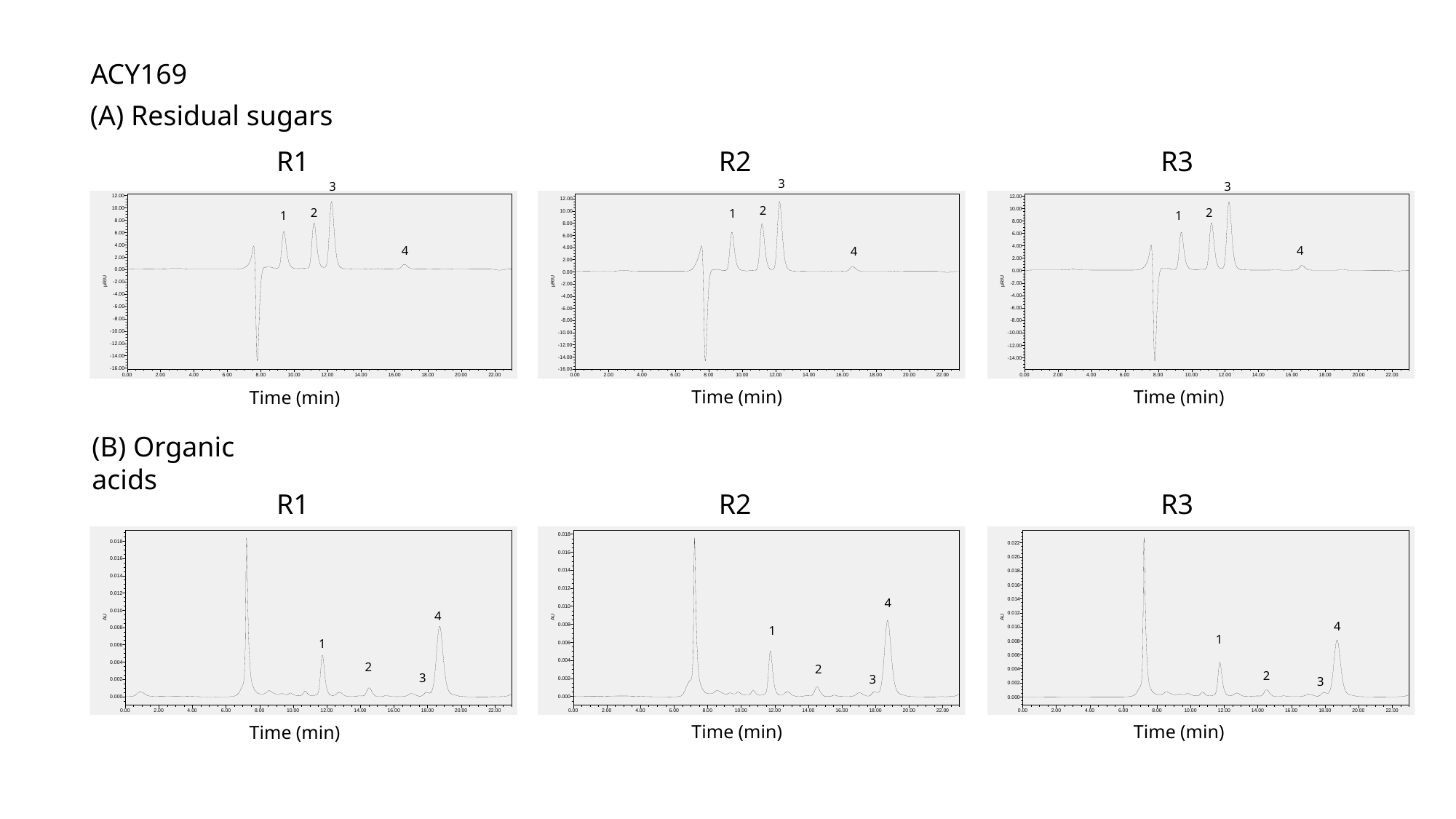

ACY169
(A) Residual sugars
R1
R2
R3
3
3
3
2
2
2
1
1
1
4
4
4
Time (min)
Time (min)
Time (min)
(B) Organic acids
R1
R2
R3
4
4
4
1
1
1
2
2
2
3
3
3
Time (min)
Time (min)
Time (min)

## Slide 21
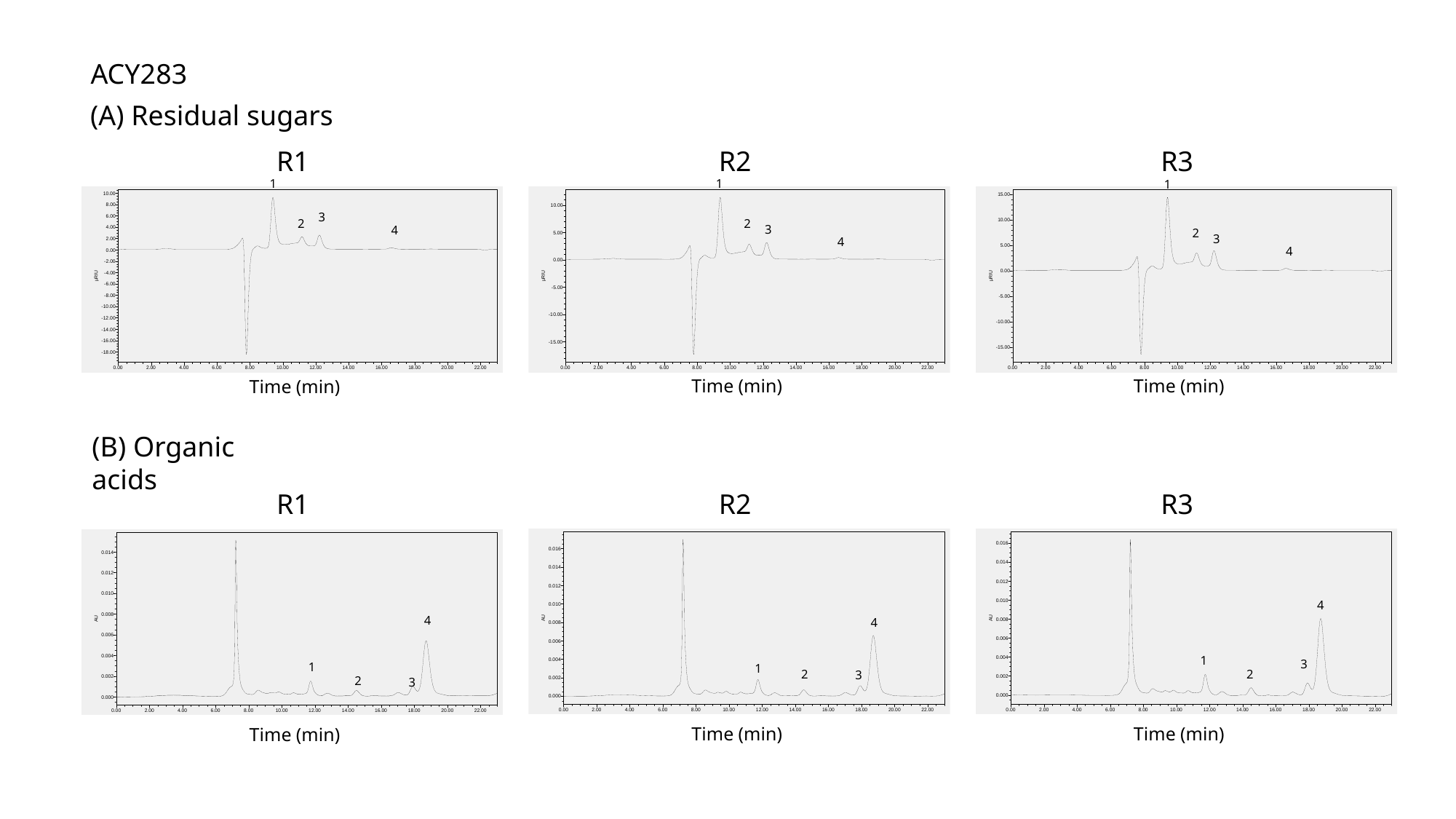

ACY283
(A) Residual sugars
R1
R2
R3
1
1
1
3
2
2
3
4
2
3
4
4
Time (min)
Time (min)
Time (min)
(B) Organic acids
R1
R2
R3
4
4
4
1
3
1
1
2
2
3
2
3
Time (min)
Time (min)
Time (min)

## Slide 22
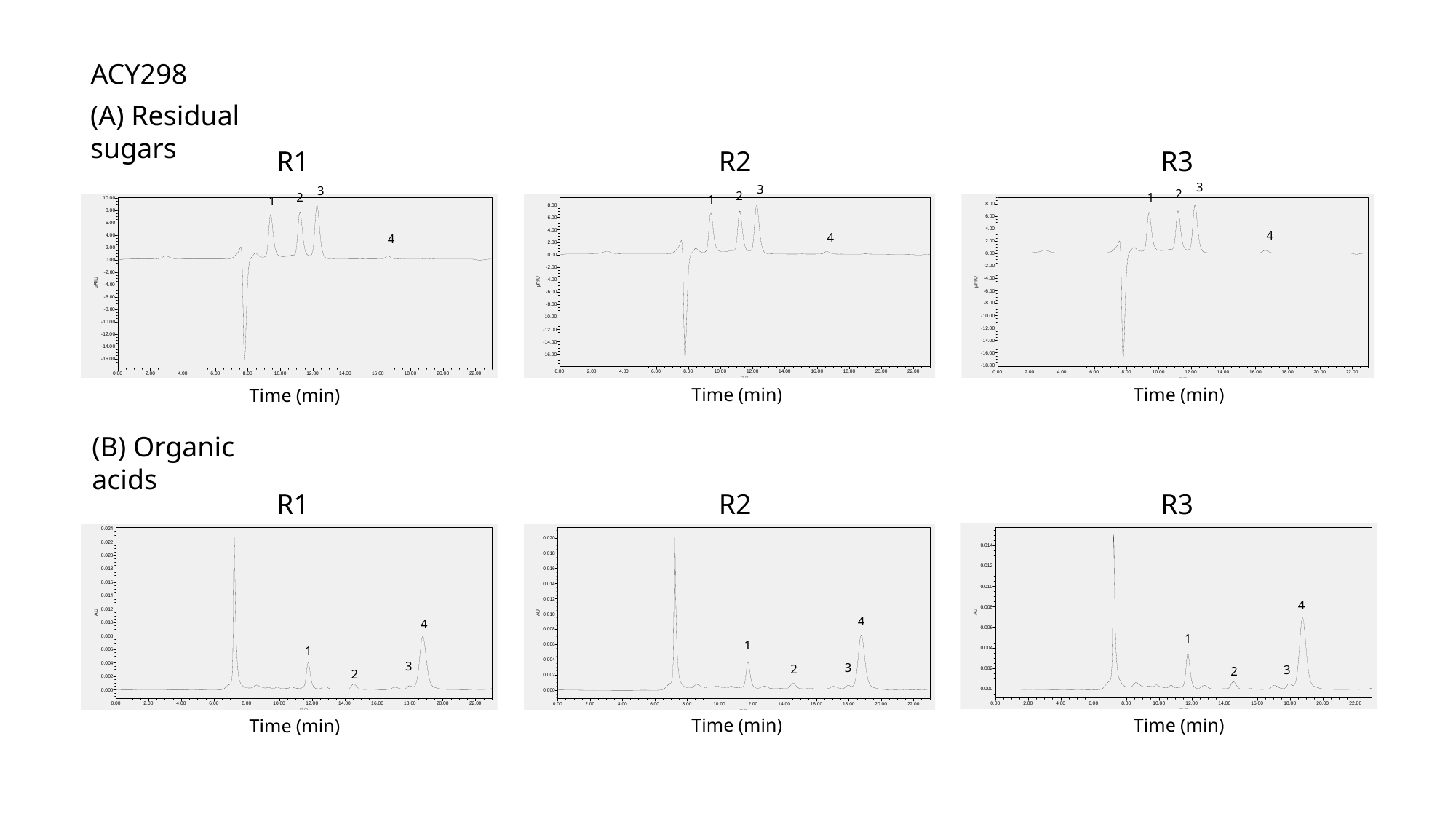

ACY298
(A) Residual sugars
R1
R2
R3
3
3
3
2
2
2
1
1
1
4
4
4
Time (min)
Time (min)
Time (min)
(B) Organic acids
R1
R2
R3
4
4
4
1
1
1
3
3
2
3
2
2
Time (min)
Time (min)
Time (min)
